# Human serum-derived protein removes the need for coating in defined human pluripotent stem cell culture

**DOI:** 10.1038/ncomms12170

**Published:** 2016-07-13

**Authors:** Sara Pijuan-Galitó, Christoffer Tamm, Jens Schuster, Maria Sobol, Lars Forsberg, Catherine L. R. Merry, Cecilia Annerén

**Affiliations:** 1 Department of Medical Biochemistry and Microbiology, Box 582, Uppsala University, 751 23 Uppsala, Sweden; 2 Department of Immunology, Genetics and Pathology and Science for Life Laboratory, Box 815, Uppsala University, 751 08 Uppsala, Sweden; 3 Stem Cell Glycobiology Group, Wolfson Centre for Stem Cells, Tissue Engineering & Modelling Room A59, University of Nottingham, NG7 2RD Nottingham, UK; 4 GE Healthcare Bio-Sciences AB, Björkgatan 30, 751 84 Uppsala, Sweden

## Abstract

Reliable, scalable and time-efficient culture methods are required to fully realize the clinical and industrial applications of human pluripotent stem (hPS) cells. Here we present a completely defined, xeno-free medium that supports long-term propagation of hPS cells on uncoated tissue culture plastic. The medium consists of the Essential 8 (E8) formulation supplemented with inter-α-inhibitor (IαI), a human serum-derived protein, recently demonstrated to activate key pluripotency pathways in mouse PS cells. IαI efficiently induces attachment and long-term growth of both embryonic and induced hPS cell lines when added as a soluble protein to the medium at seeding. IαI supplementation efficiently supports adaptation of feeder-dependent hPS cells to xeno-free conditions, clonal growth as well as single-cell survival in the absence of Rho-associated kinase inhibitor (ROCKi). This time-efficient and simplified culture method paves the way for large-scale, high-throughput hPS cell culture, and will be valuable for both basic research and commercial applications.

## Abstract (web summary)

Improved culture methods are needed to reliably grow human pluripotent stem cells (hPSCs) on a large scale. Here, the authors identify a xeno-free medium with a supplement of Inter-α-inhibitor that supports long-term propagation and improved single-cell passaging of hPSCs on uncoated plastic.

## Graphical abstract

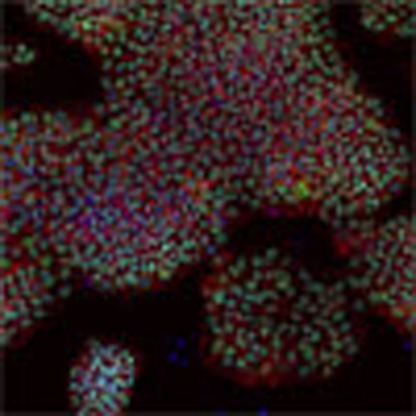

Human pluripotent stem (hPS) cells, including human embryonic stem cells (hES cells) and induced pluripotent stem cells (hiPS cells), can self-renew indefinitely while retaining the capacity to differentiate into any somatic cell type. They therefore have great potential in various applications including basic developmental research, drug/toxicity screening and cell-based therapeutics^1^. The complex matrix requirements of hPS cells, which make up the hPS cell ‘niche', are well documented and traditionally hPS cell expansion has necessitated culture on feeder cells and serum-containing media^2^. However, incompatibility of these complex, ill-defined conditions with pharmacological and medical applications has driven the development of alternative strategies combining defined media with improved surfaces. Solutions typically include surface immobilization of cell-binding motifs, such as integrin-binding proteins^3^,^4^, short peptides derived from vitronectin (VN), laminin (LN)^5^,^6^, glycosaminoglycan (GAG)-binding peptides^6^,^7^ and synthetic polymers^8^,^9^. Current novel approaches use high-throughput combinatorial arrays to discover fully synthetic alternatives^10^. However, to date, these methods have not been widely implemented, being either too expensive or lacking the required reproducibility, leaving feeder cells or Matrigel^11^ in widespread use. Moreover, many hPS cell lines have also proven resistant to successful adaptation to feeder-free conditions. There are many hPS cell-specific issues that need to be addressed for optimal culture. Routine culture usually involves passage in small aggregates or clumps to avoid a loss of viability associated with dissociation (anoikis). The addition of ROCKi (Y-27632) to the hPS cell medium increases survival after single-cell passaging, but it is costly, particularly at scale^12^. Recent years have seen considerable efforts made to simplify and refine medium formulations, and the fully defined E8 medium is an important step forward in this regard^5^. Containing only eight components, all of them produced recombinantly, E8 is typically used together with a recombinant VN peptide which is pre-applied to the tissue culture (TC) plastic as a coating for optimal cell attachment and survival (the one used in this study is Vitronectin-XF (VN-XF), distributed by Primorigen and StemCell Technologies). The combination of E8 and VN-XF has the benefits of being xeno-free and defined. However, as shown in the present study, this method does not robustly support cloning and single-cell passaging unless cells are pre-treated for several hours with ROCKi. Large-scale and automated systems of the future will require affordable reagents available in large quantities, and simplified methodologies that can support efficient and reliable single-cell passaging.

The IαI protein family members are present at high concentrations (0.6–1.2 mg ml^−1^) in human serum^13^. Inter-α-inhibitor (IαI), which is the most common family member, consists of two heavy chains (HC1 and HC2) and a bikunin (Bk) domain linked together by chondroitin sulphate^13^,^14^. The IαI complex has been associated to inflammation processes, hepatitis, cancer and even kidney diseases^15^,^16^,^17^,^18^,^19^. Traditionally, IαI has been described as an extracellular matrix (ECM) component. The HCs are the only proteins known to covalently bind the ECM GAG hyaluronan (HA), while Bk when released from the HCs acts as a serine protease inhibitor^14^,^20^,^21^. However, recent reports demonstrate that the HC domains can additionally induce cell signalling^20^,^22^,^23^,^24^. Indeed, we recently showed that IαI (specifically its HC2 domain) activates the Yes/Yes-associated protein (YAP)/TEAD transcription factor pathway and induces expression of Oct4 and Nanog in mouse ES (mES) cells^24^.

In the present study we demonstrate, for the first time, that IαI and its HC2 domain promote attachment and survival of hPS cells. Moreover, we describe a new, simplified and time-efficient cell culture method based on the E8 medium supplemented with soluble IαI (herein called E8:IαI). This method requires no surface coating and supports long-term propagation of hPS cells, clonal expansion and single-cell passaging even in the absence of ROCKi.

## Results

### IαI and its HC2 domain induce hPS cell attachment

**Figure 1:**
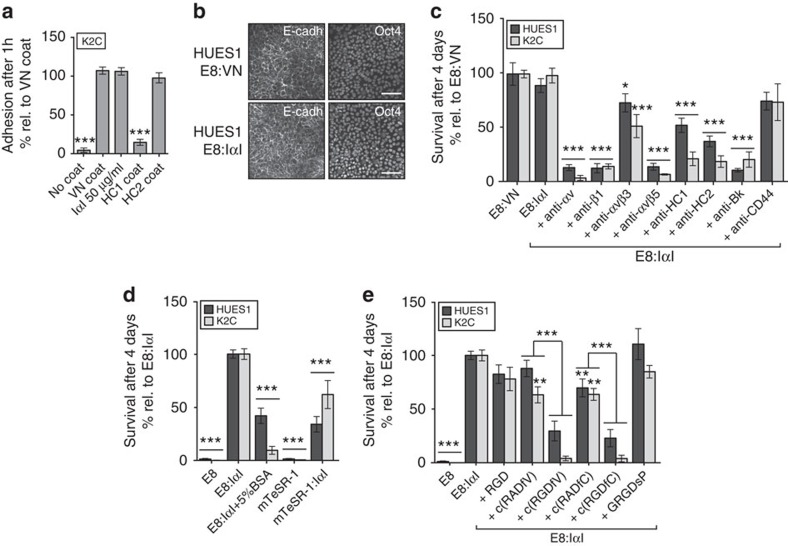
IαI and IαI domain HC2 can support attachment and survival of hPS cells. (**a**) Quantification of cell attachment by crystal violet staining 4 h after seeding of hiPS cell line K2C added to plates with 10 μg ml^−1^ VN-XF coating, 50 μg ml^−1^ IαI medium supplementation, 50 μg ml^−1^ HC1 coating or 50 μg ml^−1^ HC2 coating. (**b**) Immunofluorescence of hES cell line HUES1 on VN-XF coating or IαI supplement showing E-cadherin (left panel) and Oct4 (right panel), scale bar shows 100 μm. (**c**) Cell survival and growth assayed with crystal violet staining after 4 days of culture of HUES1 and K2C in E8 medium with IαI and different blocking antibodies, (**d**) E8 medium with or without 50 μg ml^−1^ IαI (E8:IαI), 5% BSA (w/v) in E8: IαI, and mTeSR1 medium with or without IαI; (**e**) E8 medium supplemented with IαI and IαI together with different integrin-blocking peptides. All cell-number quantification experiments were performed in triplicate over three separate experiments. Bars show mean±s.e.m. and statistical analysis over the three independent experiments. Statistical analysis indicates significant differences with *P<0.05, **P<0.001 and ***P<0.001 determined by One-way ANOVA and Dunnett's post-test. Specific comparisons were done with paired t-test and are shown by a connective line between the two sets of samples.

To study whether IαI could support stem cell attachment, K2C hiPS cells were seeded onto standard TC-treated plastic in E8 medium supplemented with 50 μg ml^−1^ IαI. The purity of the isolated IαI protein solution was thoroughly validated through silver staining and mass spectrometry analysis (Supplementary Fig. 1). Cell attachment was assessed 4 h after seeding using crystal violet staining, and VN-XF coated plates (E8:VN) were used as positive control. Figure 1a shows how IαI supplementation induces similar cell attachment levels as VN-XF coating. The separate Bk and HC1 IαI domains were unable to support attachment when added in solution to the medium (Supplementary Fig. 2; Fig. 1a). However, when HC2 (but not HC1) was pre-coated onto standard TC-treated plastic, similar attachment levels, as with IαI supplementation, were achieved 4 h after seeding (Fig. 1a). Cells seeded in IαI or on HC2-coated plates exhibited similar colony morphology and cell–cell contacts (defined by E-cadherin arrangement) as cells seeded on VN-XF (Fig. 1b; Supplementary Fig. 3). To further investigate the role of the different IαI domains in the hPS cell attachment we added antibodies against each of the three IαI domains at the seeding step. Even though the use of the separate IαI domains showed HC2 to be responsible for the hPS cell attachment, all three antibodies inhibited IαI-induced attachment regardless of which domain was being targeted, suggesting that antibody binding hinders IαI-mediated attachment when used in solution (Fig. 1c).

### Serum albumin inhibits IαI-mediated attachment

When an alternative stem cell culture medium, mTeSR1 (which contains a high concentration of bovine serum albumin (BSA, 1% w/w) and β-Mercaptoethanol, was used as basal medium for IαI, significantly lower cell attachment was achieved as compared with using E8 medium (determined by paired *t*-test). We therefore hypothesized that BSA may bind and sequester IαI, reducing its effective concentration in the medium. To test this, IαI-mediated attachment of the hES cell line HUES1 and the hiPS cell line K2C was measured in E8 medium, E8 medium supplemented with 5% w/v BSA or mTeSR1 medium. Addition of BSA to the E8 medium significantly reduced IαI-mediated attachment (determined by paired *t*-test) to similar levels as those observed when using mTeSR1 (Fig. 1d). Albumin is commonly used to block non-specific binding to plastic or other surfaces because of its rapid and wide-ranging binding capacity. To confirm that the inhibition of IαI-mediated attachment observed in the presence of albumin was not due to a competition for surface coating, we pre-coated the surface with 50 μg ml^−1^ IαI for 1 h at room temperature (RT) before seeding hPS cells in either E8 medium or mTeSR1 medium. Again, the attachment was lower in mTeSR1 as compared with the E8 medium, further confirming that albumin can indeed reduce the ability of IαI to promote cell attachment, possibly by binding to it directly and thereby blocking its effect (Supplementary Fig. 2).

### Integrins are involved in IαI-mediated attachment

The HCs contain a von Willebrand type A (vWA) domain, which has been reported to bind proteins such as VN via RGD (arginine-glycine-aspartate) motifs^25^. To assess if the observed effect of IαI supplementation was mediated by integrin engagement of RGD-motifs, a panel of RGD-blocking peptides were tested for their ability to inhibit IαI-mediated attachment (Fig. 1e). The linear RGD peptide H1830 did not have an effect, but the cyclic RGD-based, α_v_β_3_- and α_v_β_5_-blocking peptides (c(RGDfV), H-2574 (ref. 26)) and (c(RGDfC), H-7226 (ref. 27)) significantly reduced attachment as compared with their controls c(RADfV) and c(RADfC) (H-4088 and H-7232, respectively) (determined by paired *t*-test). These peptides block attachment to VN and LN, which have been shown to support hPS cell attachment and survival^4^,^5^,^7^. The GRGDsP (H-3164)-blocking peptide, which reduces attachment to fibronectin but does not interfere with VN-mediated binding, did not reduce IαI-induced attachment (all peptides were from Bachem and are described in Supplementary Table 1).

To confirm the results obtained with the RGD-blocking peptides, we used blocking antibodies against VN and LN-binding integrins together with the E8:IαI method. The antibodies were added at the seeding step and cell survival and growth was assessed after 4 days. Blocking the α_v_ and β_1_ integrin domains and the α_v_β_5_ integrin drastically affected attachment, while the α_v_β_3_-blocking antibody reduced the attachment to a lesser extent (Fig. 1c). The β_1_ integrin domain has been shown to be the main integrin domain responsible for hPS cell binding to LN^3^,^4^, while α_v_ has been described as VN binding in hPS cells when present in α_v_β_1_, α_v_β_3_ and α_v_β_5_ complexes^28^. Together, these results point towards the involvement of multiple integrin subunits in IαI-mediated attachment, in contrast to the limited number of subunits involved in binding to simple ECM-derived fragment coatings^3^.

### HA is involved in IαI-mediated attachment

HA is a non-sulphated GAG found in many matrices throughout the body where it affects multiple functions including cell migration and proliferation. HA hydrogels have also proven useful for hPS cell culture and delivery^29^. The HCs of IαI are the only proteins yet described to bind covalently to HA, and we therefore investigated if HA was involved in the IαI-mediated attachment of hPS cells. CD44 is a common HA-receptor and has been linked to cancer cell attachment and transformation following HA binding. However, we observed no inhibition of IαI-induced attachment in the presence of a CD44-blocking antibody (Fig. 1c). The involvement of GAGs in IαI-mediated attachment was further studied using a chemical inhibitor of GAG synthesis, 4-methylumbelliferone (4-MU)^30^,^31^. The hES cell line HUES1 was treated with 0.6 mM of 4-MU for 24 h before passaging and seeded onto VN-coated plates or E8:IαI, with 0.6 mM 4-MU supplementation. As shown in Supplementary Fig. 4, 4-MU treatment significantly reduced IαI-mediated attachment to ∼75% of the control samples, and this reduction was not observed for VN-mediated attachment (determined by one-way ANOVA analysis and Dunnett's post-test against negative control E8:VN). Whilst it is noted that a reduction in GAG synthesis may impact multiple aspects of cell behaviour, attachment of 4-MU-treated cells could be fully recovered by adding exogenous high molecular weight HA (HMW-HA, 2,000–2,400 KDa) therefore suggesting that HA may be involved in E8:IαI-mediated attachment, however via a non-CD44-related mechanism (determined by one-way ANOVA analysis and Dunnett's post-test against negative control E8:VN, and paired *t*-test against 4-MU-treated E8:IαI sample).

Live monitoring of the hES cell line HUES1 shows how IαI induces cell attachment and spreading over a similar timeframe and in a similar manner as the VN-XF coating (Supplementary Movie 1). Thus, IαI appears to exert a direct effect on cell attachment, similar to VN-XF coating, with attachment and spreading as concurrent events. The above data therefore point towards a complex attachment process, likely to involve several membrane receptors and extracellular ligands.

### IαI efficiently supports single-cell passaging of hPS cells

**Figure 2:**
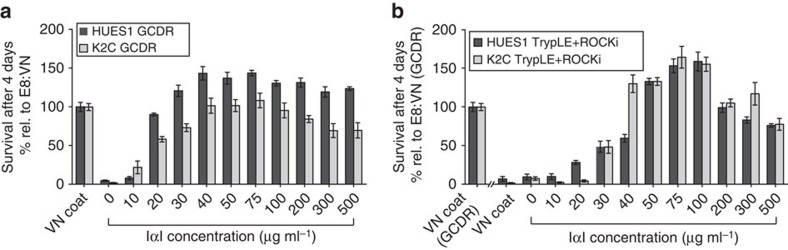
IαI attachment is concentration dependent. Dose–response curve of hPS cell lines HUES1 and K2C seeded as (**a**) small clumps (GCDR) or (**b**) single cells with ROCKi (TrypLE+10 μM ROCKi after seeding, no ROCKi pre-treatment) using increasing concentrations of IαI in E8 medium, compared with VN-XF coating. All cell-number quantification experiments were performed in triplicate over three separate experiments. Bars show mean±s.e.m.

To further evaluate IαI-induced hPS cell attachment and growth; HUES1 and K2C hPS cells were dissociated into small aggregates using a gentle enzyme-free reagent (Gentle Cell Dissociation Reagent (GCDR, Stem Cell Technologies)), or into single cells using TrypLE (a recombinant cell-dissociation enzyme, ThermoFisher) with ROCKi supplementation. The cells were then seeded using E8:VN or E8 with different IαI concentrations ranging from 0 to 500 μg ml^−1^. On day 4, cell density was assessed using crystal violet staining. As shown in Fig. 2 and Supplementary Fig. 5, 40–100 μg ml^−1^ of IαI was able to support attachment, characteristic colony morphology and positive alkaline phosphatase staining; 50 μg ml^−1^ IαI was therefore selected for all subsequent experiments (and is denoted as E8:IαI). It was also confirmed that IαI addition is only required during seeding and the initial attachment process, and is not required in subsequent medium exchanges.

**Figure 3:**
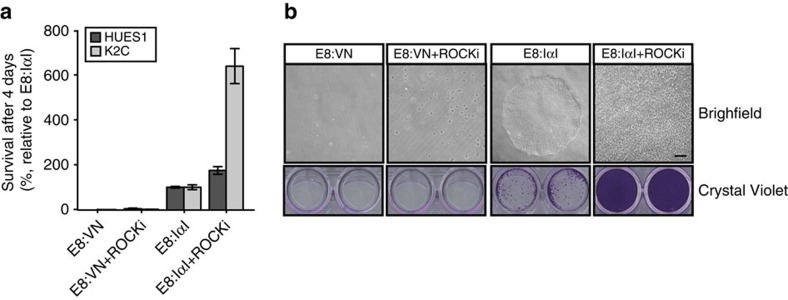
IαI increases survival after single-cell splitting. (**a**) Quantification of HUES1 and K2C cell survival and growth, using crystal violet staining, after 4 days in culture on VN-XF coat or with IαI supplementation after single-cell passaging with or without addition of 10 μM ROCKi after seeding and no ROCKi pre-treatment (**b**) representative images for HUES1 cell line, brightfield pictures on the top and overview of crystal violet on the bottom. All cell-number quantification experiments were performed in triplicate over three separate experiments. Bars show mean±s.e.m. in comparison to E8:IαI values.

When sub-culturing in small aggregates, 50 μg ml^−1^ of IαI supplementation at the seeding step supported similar viability as VN-XF coating. However, when performing single-cell splitting, almost no cells had survived after 4 days on VN-XF coating even with the addition of 10 μM ROCKi to the culture during the first 24 h after seeding. In contrast, under these conditions, IαI supplementation supported very high cell survival, with cells reaching a monolayer after 4 days. Further analysis showed that IαI was also able to support survival and colony formation of dissociated single cells in the absence of ROCKi, although the survival rate was lower than with ROCKi (Fig. 3a,b). Live monitoring of hES cells (HUES1) cultured in the absence of ROCKi showed that although the cells initially attach and spread as single cells on VN-XF coating, they eventually lift off the plate and die, while HUES1 cells seeded in the presence of IαI exhibit decreased motility after seeding but stronger cell-to-cell contact formation, and grow as adherent colonies until they are ready to be passaged (Supplementary Movie 2).

As presented above, in the absence of ROCKi pre-treatment, no hPS cell survival was observed on VN-XF coating. A 1-h pre-treatment with ROCKi is usually recommended for single-cell splitting of hPS cells to increase cell survival^12^. However, in our hands, 1-h pre-treatment resulted in inconclusive results when using the E8:VN method, even at high seeding densities. To prevent this, the ROCKi pre-treatment step was increased to 5 h. This longer pre-treatment improved the survival in E8:VN, but not to the level seen with E8:IαI. Thus, as shown in Supplementary Fig. 6a, there was a consistent higher cell density after 4 days in E8:IαI cultures when compared with VN-XF coating, from very low cell-seeding densities (25 cells per cm^2^) up to typical seeding densities (10^3^ cells per cm^2^).

### IαI protects from residual protease remaining after passage

IαI has been reported to act as protease inhibitor through its bikunin domain^32^. E8 is a low-protein medium and therefore, small traces of protease remaining in the culture medium after splitting could result in cell damage and subsequent death due to the lack of protease inhibitors. TrypLE is a recombinant protease that has been reported to be more gentle on the cell surface, and is therefore recommended for stem cell culture^33^. To investigate if the reduced cell survival observed using VN coating together with single-cell splitting is caused by remaining traces of TrypLE in the medium, we used a 5-h ROCKi pre-treatment followed by TrypLE passaging with a dilution and centrifugation step to remove all TrypLE remains. The cells were then seeded with and without the addition of 1% (V/V) of TrypLE. Supplementary Fig. 6b shows that while E8:IαI could support ∼100% survival of hES cell line HUES1 in 1% TrypLE, VN coating was unable to support cell survival in 1% TrypLE. These data suggest that IαI is able to partially protect against residual TrypLE in the medium after passaging.

### VN in solution does not support single-cell seeding

According to the manufacturer's recommendations, the VN peptide should be used as a coating agent in combination with E8 medium. To determine whether VN could be added in solution, similar to IαI, cells were pre-treated with ROCKi for 5 h and single cells were then seeded on either VN-coated plates or on uncoated plates in E8 medium supplemented with 10 μg ml^−1^ VN or 50 μg ml^−1^ IαI. As shown in Supplementary Fig. 6c, VN added in solution could not support attachment and survival to the same extent as IαI added in solution or coated VN, demonstrating that IαI-induced cell attachment is not only a result of rapid coating of the tissue culture plastic and that VN is only able to support attachment when used as a coating agent (Supplementary Fig. 6c).

### IαI increases clonal survival of hPS cells

Live imaging of NCL1 hES cells seeded in E8:IαI with ROCKi after single-cell splitting revealed a colony forming from one single cell, suggesting that IαI can support clonal growth (Supplementary Movie 3). We therefore assessed the cloning success rate for six different hPS cell lines (K2C, HUES1, H207, H181, NCL1 and K8F) cultured in E8:IαI or E8:VN. High cloning rates of up to 18% were achieved with E8:IαI, and the cloning efficiency was consistently higher using the E8:IαI protocol as compared with E8:VN protocol in all five hPS cell lines tested, despite using a 5 h pre-treatment with ROCKi (Table 1).

### Easy adaptation of hPS cell lines to E8:IαI

**Figure 4:**
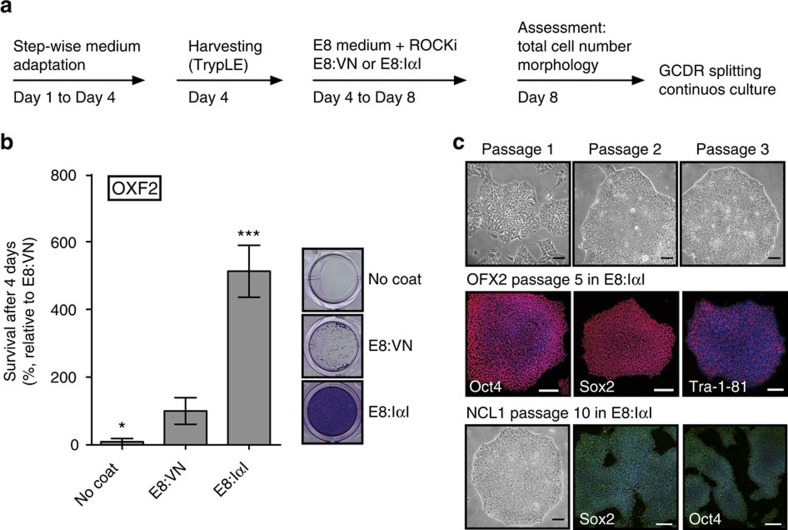
IαI achieves high-adaptation efficiency of feeder-dependent hPS cell lines. (**a**) Step-wise adaptation sequence for feeder-dependent hPS cell lines to E8:IαI and E8:VN (**b**) Crystal violet staining and quantification of OXF2 cell survival and growth 4 days after adaptation to E8:IαI or E8:VN, right panel shows representative images. (**c**) Colony morphology and immunofluorescence staining of stem cell factors Oct4, Nanog and Tra-1-81 of OXF2 after adaptation of hES cell lines OXF2 and NCL1 from feeders and serum-containing medium to E8:IαI. Scale bars show 100 μm. All cell-number quantification experiments were performed in triplicate over three separate experiments. Bars show mean±s.e.m. and statistical analysis shows significant difference with *P<0.05, **P<0.001 and ***P<0.001 (determined by One-way ANOVA and Dunnett's post-test) in comparison to E8:VN values.

To ensure that the E8:IαI protocol can support robust propagation and pluripotency of different hPS cells, six hES cell lines (HUES1, H181, H207, OXF2, NCL1, huES3-Hb9::GFP) and two hiPS cell lines (K2C and K8F), originally derived in four different laboratories, were adapted to E8:IαI. All hPS cell lines exhibited normal morphology in E8:IαI medium (Supplementary Fig. 3a). The E8:IαI protocol showed better adaptation efficiency of the hES cell line OXF2 from feeder-cell culture as compared with E8:VN. Through a simple step-wise adaptation protocol, combining E8:IαI and single-cell passaging using TrypLE dissociation and ROCKi (Fig. 4a), we achieved 100% adaptation efficiency of five different feeder-dependent hPS cell lines (OXF2, NCL1, huES3::Hb9rGFP, K2C and K8F), with approximately four-fold better yield as compared with the E8:VN protocol for OXF2 hES cells (Fig. 4b). Healthy colony formation was achieved at passage 1 and negligible differentiation was observed from passage 2 (Fig. 4c). Successful adaptation of NCL1 was particularly noteworthy since this line had, in our hands, proved incompatible with feeder-free culture. These data suggest enhanced adaptation efficiency of feeder-dependent hPS cell lines to E8:IαI. Assessment after 5–10 passages proved expected stem cell marker expression (Fig. 4c).

### IαI supports long-term propagation of hPS cells

**Figure 5:**
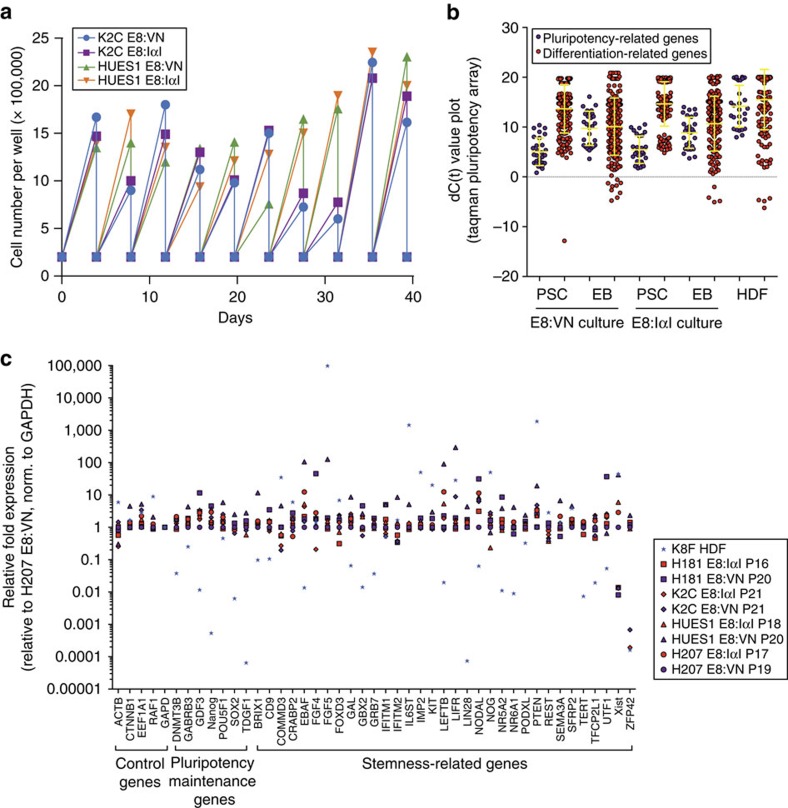
IαI maintains pluripotency and self-renewal of hPS cells in long-term culture. (**a**) Growth curve of K2C hiPS cell line and HUES1 hES cell line in either E8:VN or E8:IαI for 10 passages. (**b**) Dot-plot illustrating combined dC(t) values of pluripotency genes (purple dots) and differentiation genes (red dots) obtained using the Taqman pluripotency array on hPS cell lines HUES1, H207, H181 and K2C after 16 or more passages in E8:VN or E8:IαI (PSC) and after differentiation through EB formation (EB) compared with human dermal fibroblasts (HDF), note that higher C(t) values translate to lower expression, and average distribution is represented with yellow bars. (**c**) Relative expression values for 45 housekeeping, pluripotency and stemness genes across the four hPS cell lines after long-term passaging in E8:VN (outlined blue symbols) or E8:IαI (outlined red symbols), relative expression to H207 grown in E8:VN and normalized to GAPDH expression, and compared to human dermal fibroblast line K8F (HDF, blue stars). No statistical difference was found (determined by Two-way ANOVA and Sidak's post-test). Note that E8:IαI samples exhibit less variation in pluripotency gene expression levels as compared to the E8:VN samples.

**Figure 6:**
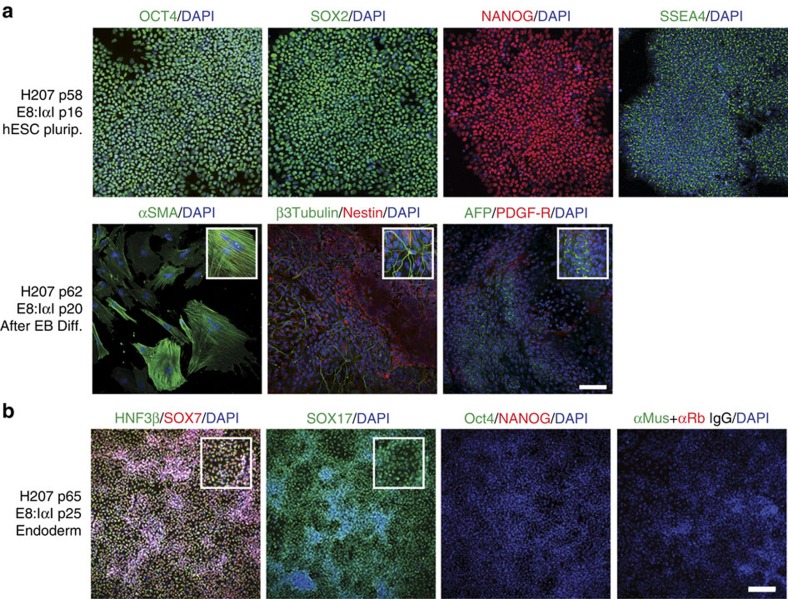
IαI maintains pluripotency and differentiation capacity in long-term culture. Immunofluorescence staining of H207 hES cell line for (**a**) stem cell markers Oct4 (green), Sox2 (green), Nanog (red) and SSEA-4 (green) after 16 passages in E8:IαI, and for the three germ layers after 4 weeks of differentiation through embryoid body (EB) formation: mesoderm with alpha smooth muscle actin (SMA, green), ectoderm with Nestin (red) and β-III-tubulin (green) and endoderm with alpha-fetoprotein (AFP, green) and PDGF-receptor (red), and (**b**) after endoderm directed differentiation for endoderm markers Sox7 (red), Sox17 (green) and HNF3β (green) and stem cell markers Oct4 (green) and Nanog (red), as well as negative control with secondary antibodies. All samples were co-stained with DAPI (Blue) for nuclei detection. Scale bars show 100 μm, white boxes show close-up images.

Three hES cell lines (HUES1, H207 and H181) and one hiPS cell line (K2C) were then cultured for 15 or more passages in E8:IαI. The E8:VN protocol was used as positive control and, for adequate comparison, cells were split in small clumps for both protocols. All cell lines grew as tight colonies with negligible signs of differentiation regardless of culture condition. E8:IαI also supported freeze-thaw cycles with survival rates after recovery similar to E8:VN. Proliferation rate was assessed for up to 40 days (10 passages) and showed similar growth rates independent of culture protocol and cell line (Fig. 5a). Immunofluorescence staining highlighted strong and specific expression of stem cell markers in all cell lines after 15 or more passages in both protocols (Fig. 6a; Supplementary Fig. 7).

The Human Stem Cell Pluripotency TaqMan array (ThermoFisher) was performed to characterize expression profiles of the four hPS cell lines after long-term propagation focusing on marker genes for pluripotency, stemness and differentiation^34^. No significant differences in transcription profiles were found between culture conditions (as determined by multiple comparisons using two-way ANOVA and Sidak's Multiple test), indicating that IαI does not induce differentiation or reduce expression of stem cell related markers (Fig. 5b,c; Supplementary Fig. 8; Supplementary Data 1). In fact, when relative expression values for stem cell markers were assessed closely, it was found that hPS cells grown in E8:IαI showed a more compact expression profile than hPS cells grown in E8:VN (Fig. 5b,c; Supplementary Fig. 8). The cells were then subjected to spontaneous differentiation by embryoid body formation and subsequent plating onto Matrigel with serum-containing medium. After 4 weeks, all hPS cell lines, independent of previous culture condition, showed successful differentiation into the three germ layers as assessed by immunofluorescence staining for specific markers (Fig. 6a; Supplementary Fig. 9a,b). TaqMan array was then performed on the differentiated hPS cell samples and again, no differences in transcriptional profiles were found between the two culture methods (Fig. 5b; Supplementary Data 1). Moreover, as shown in Supplementary Movie 4, K2C hiPS cells successfully produced beating cardiomyocytes after 40 passages in E8:IαI.

### IαI supports directed endoderm differentiation

Endoderm has been the most difficult germ layer to generate *in vitro*^35^. To investigate if IαI could also support directed differentiation protocols for hPS cells, we combined the STEMDiff Definitive Endoderm Kit (TeSR-E8 optimized, StemCell Technologies) with IαI. Briefly, four hPS cell lines grown in E8:IαI for 10 or more passages were pre-conditioned to endoderm before being split as single cells and seeded using IαI. All four hPS cell lines attached normally and survived throughout the five-day differentiation protocol. The results showed that cells cultured in E8:IαI can be readily induced to differentiate into endoderm, generating a monolayer of endoderm precursor cells, positive for the key early endoderm markers Sox7, Sox17 and HNF3β (ref. 36; Fig. 6b; Supplementary Fig. 10).

### IαI does not increase genetic abnormalities

To ensure that IαI does not increase the level of mutations or chromosomal abnormalities after long-term culture when compared with current methods, all hPS cell lines were subjected to G-banding and the Human OmniExpressExome array (Illumina, SNP&SEQ Technology Platform, Uppsala University). Single-nucleotide polymorphism (SNP) array data were analysed both by total number of copy-number (CN) calls, and number of shared CN calls between early and later passage (Supplementary Tables 3 and 4; Supplementary Fig. 11). G-banding analysis showed few but equal amounts of abnormalities in both culture methods (Supplementary Table 2; Supplementary Fig. 12). Further analysis done by using SNP-array data indicates that the CN profiles changed during culturing regardless of protocol used and cell line examined and that the two examined protocols perform equally well in retaining genetic profiles (Supplementary Table 3). A stricter CN analysis was also performed by comparing the number of shared CN calls between early and late passages in each of the four cell lines under the two protocols and for all 123 CN calls (Supplementary Table 4). Overall, we found no difference in the number of genetic alterations detected (Wilcoxon rank-sum-test; *W*=10.5, *P*=0.8082). No increase in genetic abnormalities was observed in E8:IαI as compared with E8:VN and the starting passages, suggesting that IαI does not increase the risk of genetic aberrations or selection for advantageous mutations in long-term culture. Even though the presence of abnormalities in all conditions may seem alarming, the hPS cell lines used in this study were obtained at high passages, and it has been reported that hPS cell lines can develop abnormalities after long-term culture *in vitro*^37^. Indeed, a previous large-scale analysis of 125 independent hPS cell lines from 38 different laboratories reported that small variations similar to the ones present in this study could be found in all samples, and documented that these are consistent with previous structural variants studies made on human populations, therefore dependent on their source genome and not from their *in vitro* culture^38^.

## Discussion

The last few years have seen a marked refinement of hPS cell culture methods, enabling the use of better defined culture conditions as well as simplified handling protocols, essential for the widespread use of hPS cells. The potential of hPS cells as renewable cell sources for drug toxicity screening, human cell therapy and regenerative medicine has rendered traditional cell culture methods, based on ill-defined components, unsuitable^39^. For this purpose, several defined surfaces have been developed to improve hPS cell derivation and expansion^40^,^41^. However, none of the currently available methods fulfil all requirements for simple, robust, cost-efficient and safe production of hPS cells. The combination of E8 medium with VN-XF^5^ coating is both defined and animal-component free, and has been proven to successfully support long-term culture of hPS cells when passaged as small clumps. However, this method requires a surface pre-coating step and, as shown in the present work, is inefficient when hPS cells are passaged as single cells.

In this paper we present IαI as a protein able to induce attachment of hPS cells onto plastic without the need for a pre-coating step. This is the first time that IαI has been reported to induce attachment in *in vitro* culture, particularly remarkable considering the extensive and numerous efforts to identify substrates for hPS cell culture. During this study we discovered that high concentrations of albumin in culture medium reduce IαI-induced attachment to ∼50%. These results suggest that albumin binds IαI and reduces its ability to induce cell attachment, both when present as a soluble factor and a pre-coated agent. This is possibly the reason why this is the first study linking IαI to cell attachment, as the majority of traditional cell culture media contain high concentrations of albumin.

IαI is a relatively large protein that consists of three distinct globular domains: Bk, HC1 and HC2. We found that antibodies targeted against any of the IαI domains could block IαI-induced attachment of hPS cells when IαI was used in solution. However, the only domain that could support hPS cell growth on its own was HC2, and only when HC2 was used as a coating agent. These results concur with our previous report showing that the HC2 domain of IαI, but not the HC1 domain, can activate pluripotency-related transcription in mES cells^24^. It has also been reported that transfer of the HC domains to HA requires the HC2 domain to be present, as part of the IαI molecule or as a separate domain (for example, HC2-Bk)^20^,^23^, confirming the distinct biological activity of the HCs. While HC1 and HC2 share functional domains (a vault protein inter-α-trypsin (VIT) domain and a vWA domain), outside of these regions there is considerable heterogeneity, and overall, human HC1 and HC2 only share 39% amino acid identity.

While little is known about the function of the VIT domain, the vWA domain has been reported to bind many proteins via RGD domains, including VN^25^. Thus, to assess if the IαI-induced attachment was integrin mediated, we used RGD-blocking peptides as well as integrin-blocking antibodies and found that multiple integrin subunits were likely to be involved in the process, pointing to a more complex interaction than the traditional integrin-binding protein attachment of VN- and LN-based coatings. Since the HCs of IαI are the only proteins reported to bind covalently to HA, we also investigated the potential role for HA in IαI-mediated attachment. Blocking the HA receptor CD44 did not have any effect on IαI-induced attachment. However, reducing GAG synthesis using an inhibitor decreased IαI-mediated attachment, indicating that HA may play a role IαI-mediated attachment. Overall, our results point to IαI-induced attachment being the result of a synergistic combination of several extracellular molecules and surface receptors. Indeed, IαI has been reported to interact with several extracellular molecules, including HA^20^, integrin-binding proteins such as VN^25^, and the complement system^42^. *In vivo*, many different processes have been linked to IαI, including the cumulus formation of the oocyte^43^, fibrosis and angiogenesis^22^, tissue repair^25^, amniotic membrane formation^44^,^45^, arthritis^46^ and others. IαI is proving to be a very versatile protein with a wide variety of functions, which most likely depend on the different partners it can interact with in each situation. Clearly, further study will be needed to completely unravel the mechanism underpinning IαI-mediated attachment.

In this study we present the first hPS cell culture protocol that overcomes the need for specialized surface coating or functionalization, while still being defined and animal-component free. This method is highly time efficient, as coating is a time-consuming step in hPS cell culture methods. We demonstrate that E8:IαI improves single-cell passaging and cloning efficiency, without the need for a lengthy ROCKi pre-treatment, and can protect from residual protease, thus making automated handling simpler and enabling cell-number control and high-throughput experimental design. Long-term propagation and characterization of four different hPS cell lines and successful adaptation of a total of eight different hPS cells lines prove that this culture method is reliable and robust. Moreover, we have shown that IαI can support a directed differentiation protocol. E8:IαI can also support freeze-thaw cycles in the same manner as commercially available methods. The IαI used in this study was derived from a side fraction of human plasma used for commercial factor IX production, with purity above 95%, as described previously^14^,^21^,^24^. IαI has a long shelf-life and can potentially be purified and prepared in large quantities from human plasma at GMP grade and at a reasonable cost. Future development will also investigate on the possibility of using recombinant IαI, or parts thereof. In conclusion, this simple method offers a reliable and cost-efficient solution to the current need for defined hPS cell culture conditions and has great potential to transform research, pharmacological and clinical application of hPS cells and their derivatives.

## Methods

### Materials and cell lines

Cells: hES cell lines H181, H207 (kindly provided by Dr Hovatta from Karolinska Institutet, Stockhom), HUES1 and HUES3-Hb9::GFP (kindly provided Dr Melton and Dr, Eggan from Harvard Stem Cell Institute and Dr. Koslova from Uppsala University), OXF2 and NCL1 (UK Stem Cell Bank), and hiPS cell lines K2C and K8F (generated by Dr Schuster, Uppsala University). Primary antibodies: mouse anti-Oct4 (POUF5F1) cl. 7F9.2 (Millipore), rabbit anti-Nanog (ab109250, Abcam), mouse anti-Sox2 (ab171380, Abcam), mouse anti-SSEA-4 (MAB4304, Millipore), mouse anti-Tra-1-81 (MAB4381, Millipore), mouse anti-Tra-1-60 (MAB4360, Millipore), mouse anti-α-Fetoprotein (AFP) receptor (MAB4086, Millipore), rabbit anti-PDGFβ receptor (sc-432, Santa Cruz Biotechnology), mouse anti-Neuron specific β-III-tubulin (MAB1195, R&D), rabbit anti-E-cadherin (#3195, Cell Signaling Technology), mouse anti-α-Smooth Muscle Actin (αSMA) (CBL171, Millipore), rabbit anti-nestin (ab105389, Abcam), mouse anti-HNF3β (sc-101060, Santa Cruz Biotechnology), mouse anti-Sox17 (SAB3300046, Sigma), rabbit anti-Sox7 (HPA009065, Sigma). Blocking antibodies used were: mAB13 (ref. 47) anti-β1 (kindly provided by Dr. Kenneth Yamada, NIDCR, USA), anti-αv L230 (ab94704, Abcam), anti-αvβ3 (MAB1976, Millipore), anti-αvβ5 (ab24694, Abcam), anti-ITIH1 K-16 (sc-33944, Santa Cruz Biotechnology), anti-ITIH2 N-17 (sc-21974, Santa Cruz Biotechnology), anti-Bk P-20 (sc-21597, Santa Cruz Biotechnology), rat anti-CD44 KM114 (kindly provided by Dr. Paraskevi Heldin, Uppsala University). Secondary antibodies: Alexa Fluor 488 goat anti-mouse (ThermoFisher), Alexa Fluor 555 goat anti-rabbit (ThermoFisher). Other reagents: Hoechst 33342 (ThermoFisher), Matrigel™ Matrix hES-qualified (Corning), RGD-based blocking peptides (all from Bachem, described in Supplementary Table 1), Y-27632 (ROCK inhibitor, ROCKi, StemCell Technologies), 25% hES-qualified BSA (Invitrogen), high molecular weight HA 2,000–2,400 kDa (HMW-HA, Cat No. 73641, Sigma-Aldrich), and 4-Methylumbelliferone (4-MU, M1381, Sigma).

### Cell culture

The hPS cell starting cultures were on Matrigel coating (Corning) in mTeSR1 medium (StemCell Technologies)^11^ or on feeder cells (gamma-irradiated MEFs or CRL-2429 HFFs) in 20% KOSR and 8 ng ml^−1^ bFGF supplemented KO-DMEM^48^. Continuous culture on VN-XF and E8 medium was performed following manufacturer's instructions. Briefly, vitronectin coating was performed by diluting VN-XF (StemCell Technologies #07180) in Dilution Buffer (StemCell Technologies #07183) to a final concentration of 10 μg ml^−1^, then immediately using the solution to coat non-tissue culture-treated cultureware, gently mixing the solution by rocking. The solution was allowed to coat for at least 1 h at RT or overnight (ON) at 4 °C. The coated well was washed once with the dilution buffer before use. The hPS cells were harversted using the Gentle Cell Dissociation Reagent (GCDR, StemCell Technologies #07174), the E8 medium was removed and the appropriate volume of GCDR was added and incubated for 5–8 min at RT. Then the solution was removed and the appropriate volume of E8 medium added, the colonies were scrapped using a cell lifter (Corning, CLS3008) and broken down by pipetting up and down 4-5 times. After that the cells were seeded using a dilution according to the initial density, typically ranging from 1:4 to 1:10.

### Culture of hPS cells in E8:IαI

For the continuous culture of hPS cells in E8:IαI the cells were grown until appropriate size before splitting using GCDR (StemCell Technologies #07174). Briefly, the medium was removed; the appropriate volume of GCDR was added to the wells and incubated at RT for 5–8 min. The GCDR solution was removed and the appropriate volume of E8 medium was added, the colonies were then harvested using a cell lifter (Corning, 900020) and broken down by pipetting up and down 4–5 times. The wells were split 1:4 to 1:10 (depending on starting cell density), fresh E8 medium together with the hPS cells was added into a new, uncoated, tissue culture-treated plastic (Corning). Finally, 50 μg ml^−1^ IαI was added into the well and mixed by gentle rocking. The culture medium was changed daily using standard E8 medium. IαI supplementation was not necessary after the initial seeding step.

### Adaptation of feeder-dependent PS cells to E8:IαI

The hPS cells cultured on feeder cells were adapted to E8 medium using a 4-day step-wise adaptation from the original medium to the E8 medium. On day 4, the cells were harvested using TrypLE and then seeded in E8 medium supplemented with 10 μM Y-27632 onto tissue culture-treated plastic with 50 μg ml^−1^ IαI supplementation or onto VN-XF coated surfaces, cell density used was approximately 5 × 10^3^ cell per cm^2^. The medium was then changed daily until day 4, when cells were passaged or assessed for total cell number. After the first passage, continuous culture passaging was done using GCDR.

### Growth-rate assessment

The hPS cell lines were split for routine culture using a GCDR and collected using a cell lifter. For the growth-rate assessment, duplicates were kept for every condition. One well was passaged with GCDR for continued culture, while the other was passaged with TrypLE for cell-number measurement with a TCP counter (Bio-Rad).

### Cryopreservation of hPS cells

The cells were harvested using GCDR and a cell lifter. The freezing medium used was mFreezer or FreSR-S (StemCell Technologies) and the final cell concentration ranged from 4-10 × 10^6^ cells per ml. Cell solution (1 ml) was used per cryovial, the cells were then transferred to a −80 °C freezer for a slow-cooling using an isopropanol freezing container. After 24 h, the cryovials were transferred to permanent storage in vapour phase liquid nitrogen.

### Differentiation

Embryoid body (EB) formation was performed using the aggrewell system (StemCell Technologies), harvesting the cells using TrypLE with 1-h ROCKi pre-treatment and seeding an average of 2,000 cells per EB and with ROCKi addition. The EBs were then kept in GMEM 20% FBS and suspension for 2 weeks and then plated onto Matrigel (Corning) or Synthemax (vitronectin, Corning) for 2 more weeks. Beating cardiomyocytes areas were filmed after 3–4 weeks of growth using a light-phase microscope coupled to a NikonE990 camera.

### Purification of human IαI

A side fraction from the commercial manufacturing of factor IX (Pharmacia-Upjohn, Stockholm, Sweden) was fractionated using gel filtration chromatography on a HiPrep 26/60 Sephacryl S-400 HR column using the ÄKTA automated chromatography system (both from GE Healthcare). This process generates more than 95% pure IαI. For the release of the heavy chains, a solution of 1 mg ml^−1^ IαI in PBS was subjected to 0.05 M NaOH treatment for 15 min at RT and then dialyzed against 20 mM sodium phosphate pH 7.6 and subsequently applied to an anion-exchange chromatography for separation using the ÄKTA system (MonoQ 5/50 GL; GE Healthcare)^49^. Fractions were analysed in 8% acrylamide SDS–PAGE gels and Coomassie Brilliant Blue staining. Unless specified otherwise, protein concentrations were determined by UV measurements and using the absorbance coefficients for the protein moieties of IαI, HC1 and HC2 obtained from Bloom *et al.*^14^ The protein solutions were concentrated and stored at −20 °C.

### Crystal violet attachment assay

The hPS cells were collected with either with GCDR or TrypLE. Approximately 5 × 10^3^ cell per cm^2^ were seeded, unless specified otherwise. For single-cell splitting, 4–5 h of 10 μM ROCKi pre-treatment was used (unless stated otherwise) and the dissociation reagent was TrypLE (ThermoFisher). For single-cell splitting 10μM ROCKi was also added for the first 24 h after seeding, unless specified otherwise^12^. After the specified time for attachment and/or growth, total cell number was assessed^50^. Briefly, the plates were washed twice with PBS to remove non-attached cells and fixed using ice-cold methanol for 10 min, and subsequently stained with 0.4% w/v crystal violet in methanol for 5 min. The crystal violet was then thoroughly washed away using distilled water, and the plates were dried before adding 175μl of 0.1% SDS solution per 24-well plate well. After 1–2 h on a tilting table at RT, quantification was performed measuring optical density at 570 nm with Multiscan PLUS (Labsystems). All cell-number quantification experiments were performed in triplicates, with three separate independent experiments performed, unless specified otherwise. The samples were also periodically randomized so as to ensure no position-related variations.

### Attachment blocking

The hPS cells were harvested as small clumps using GCDR and seeded at 5 × 10^3^ cells per cm^2^ in E8 supplemented with 50 μg ml^−1^ IαI (E8:IαI) with the addition of each antibody at 5 μg ml^−1^ for the integrin-blocking antibodies, and 20 μg ml^−1^ for the IαI-binding antibodies for the first 24 h. The RGD-blocking peptides were also added at the seeding step at a concentration of 25 μM (9–15 μg ml^−1^). The cells were cultured for 4 days before cell survival and growth was assessed using crystal violet staining.

### GAG synthesis inhibition

The inhibitor 4-Methylumbelliferone (4-MU) was used to inhibit GAG synthesis in the hPS cells to assess its effect of on the IαI-mediated attachment. Briefly, hPS cells were treated for 24 h in E8 medium supplemented with 0.6 mM 4-MU or with 6μl MeOH as a negative control. This concentration has been reported to decrease HA synthesis without inducing apoptosis in cancer cell lines^31^. After treatment the cells were harvested as small clumps using GCDR and seeded onto VN coating or with IαI, with the addition of MeOH or 4-MU for the first 24 h after seeding. Moreover, in the 4-MU-treated samples 100 μg ml^−1^ HMW-HA was also added to assess the effect of exogenous HA.

### Directed endoderm differentiation

Directed endoderm differentiation was performed using the STEMDiff Definitive Endoderm Kit (TeSR-E8 optimized, StemCell Technologies), according to the manufacturer's instructions. Four hPS cell lines cultured on E8:IαI conditions were grown for 4 days. At day four, the medium was changed to Pre-differentiated supplemented E8 medium and used for daily exchanges for 48 h. The cells were then washed with PBS and split using GCDR and thorough pipetting to achieve a single-cell suspension. The single suspension was centrifuged and resuspended in E8 medium supplemented with IαI and pre-differentiation reagent, and seeded at 2 × 10^5^ cells per cm^2^. After 24 h, the medium was changed to StemDiff Definitive Endoderm Basal medium supplemented with reagents A and B. On day 2, the medium was changed into StemDiff Definitive Endoderm Basal medium supplemented with reagent B, and this medium was used to exchange daily until day 5. The cells were then fixed and used for immunocytochemistry analysis.

### Immunocytochemistry

Cells were fixed with cold 4% paraformaldehyde (Sigma) for 15 min and then blocked with 0.5% BSA and 0.3% Triton X-100 in PBS (all from Sigma). The primary antibodies anti-Oct4 (1:300), anti-Nanog (1:100), anti-Sox2 (1:100), anti-SSEA-4 (1:200), anti-Tra-1-60 (1:100) and anti-Tra-1-81 (1:100), anti-βIII-Tubulin (1:100), anti-Nestin (1:200), anti-αSMA (1:200), anti-AFP (1:50), anti-PDGFβ receptor (1:50), anti-E-cadherin (1:100), anti-Sox7 (1:50), anti-Sox17 (1:50), anti-HNF3β (1:100), were added to the cells and incubated in a humid chamber at 4 °C overnight, rinsed with PBS, and subsequently incubated with Alexa Fluor 555 anti-rabbit (1:500) and/or Alexa Fluor 488 anti-mouse (1:500) for 60 min at room temperature. After rinsing with PBS and co-staining with Hoechst 33342, coverslips were mounted using fluoromount (Sigma), and analysed with inverted confocal fluorescent microscopy (LSM700, Zeiss). All images were uniformly processed using Adobe Photoshop CS6 (Version 13.0.6 x64).

### Human stem cell pluripotency taqman array

Total RNA was extracted using an RNeasy Mini kit (Qiagen) following manufacturer's instructions. An RNA nano6000 chip and a Bioanalyser2100 (Agilent) were used to assay RNA quality, and only samples with an RNA integrity number (RIN) ≥8 were selected for the array. cDNA was prepared from 1 μg of total RNA using iScript (Bio-Rad) according to manufacturer's instructions, and then mixed with TaqMan Gene Expression Master Mix and loaded onto a TaqMan Human Stem Cell Pluripotency Low Density Array (TLDA, ThermoFisher). The arrays were run on a 7900HT Real-Time PCR System (Applied Biosystems). The data were processed with RQ Manager 1.2.1 software and relative expression (dC(t)) was calculated using GAPDH as a control and used to compare across all cell lines using the statistical programme GraphPad Prism 6. The hierarchical cluster analysis was performed by Genesis^51^. All arrays were run at the SciLifeLab Genome facility (Uppsala, Sweden).

### G-banding karyotype

Cultures of up to 70–80% confluency were treated with 100 ng ml^−1^ colcemide (KaryoMax colcemide Solution (10 μg ml^−1^), Gibco) overnight, and harvested using TrypLE (ThermoFisher). The cells were subsequently treated with freshly prepared hypotonic solution (75 mM KCl, Sigma) for 30 min at 37 °C followed by a triple fixator treatment (3:1 Methanol:Acetic acid, both by Sigma) for 20 min each. The dropping of fixed cell suspension on cold wet microscope slides was used to achieve metaphases, which were subsequently processed using Trypsin (Sigma) and stained with Giemsa staining solution (Sigma) for G-banding analysis^52^. For each sample, twenty metaphases were analysed with IKAROS-software (MetaSystems). Description and specification of chromosomal aberrations was performed following guidelines from ISCN 2013.

### SNP array and genotyping

SNP genotyping with Illumina HumanOmniExpressExome-array version 8v1-2_A, encompassing >964.000 single-nucleotide polymorphism (SNP)-markers, was performed by the SNP&SEQ technology Platform in Uppsala (www.genotyping.se), according to the manufacturer's instructions. Each of the four hPS cell lines (that is, H181, H207, HUES1 and K2C) were analysed in three separate SNP-array experiments. Total DNA was extracted from cell culture growth at 60-70% confluence using the DNeasy Blood & Tissue Kit (Cat. No. 69504, Qiagen). DNA concentration and purity was determined by ultraviolet measurement. A first analysis was performed after 2–5 passages of growth for initial reference, and subsequently, two analyses were performed per cell line after 16–21 passages in E8:VN or E8:IαI culture. The CN calls of the autosomal chromosomes were illustrated using the Nexus software.

### Copy-number analysis

Illumina output files were analysed using Nexus-Copy-Number version 7.5 (BioDiscovery, CA, USA). Linear systematic correction and the ‘SNP-FASST2 segmentation' algorithm for CN-calling with default calling parameters was applied. A first CN analysis was performed on the autosomal chromosomes for each cell line after 2–5 passages. Strict CN-calling criteria (100% overlap of CN calls and *P*<0.05 in Nexus software) were applied. After this, each of the four cell lines was grown to 16–21 passages, applying either the E8:IαI or the E8:VN protocol. Subsequently, new CN-analyses using the same settings in Nexus software was performed and the results were compared to the CN calls from earlier. The number of shared CN calls between the early and later passages in each of the four cell lines was used to quantify changes introduced during culturing within each cell line under the E8:IαI and E8:VN protocols, respectively. We used Wilcoxon rank-sum-test in the statistical software R v3 to test for differences in the observed number of shared CN calls from the E8:IαI and E8:VN protocols.

### Clonal assay and MTT Tetrazolium assessment

The starting hPS cells were pre-treated for 4–5 h with 10 μM Y-27632 before harvesting them using TrypLE (ThermoFisher). Briefly, 1 ml TrypLE was added to a pre-treated 6-well plate well of hPS cells ready to passage. The cells were incubated with TrypLE for 8 min before removal and addition of 1 ml E8 medium with 10 μM ROCKi, the cells were harvested with a cell lifter. Cell clumps were broken down by pipetting and the solution was passed through a 40 μm pore cell strainer to ensure single-cell suspension (StemCell Technologies). The cells were then counted using the TCP counter (Bio-Rad). After that the cells were diluted down to 100 cells in 10 ml of E8 medium with 10μM ROCKi, mixed and seeded on VN coating or with IαI supplementation in 100 μl per each of 100 wells (50 wells per 96-well plate) so as to achieve approximately one cell per well. After two weeks the survival and formation of colonies was assayed visually and through MTT assay. Briefly, MTT (3-(4,5-dimethylthiazol-2-yl)-2,5-diphenyltetrazolium bromide) tetrazolium was dissolved in E8 medium at a final concentration of 0.5 mg ml^−1^, the solution was filtered and added to the wells from the clonal assay and incubated at 37 °C and 5% CO_2_ for 3 h. After confirming the staining of the existing colonies, the MTT solution was removed and 20% SDS was added. The plates were incubated O/N and the absorbance was read at 570 and 630 nm. This is a measure for viable cells. Wells with more than one colony were removed from the final result summary.

### Mass spectrometry analysis

Two samples of IαI working solution (purified separately) were mixed with β-mercaptoethanol and run through a 12% acrylamide SDS–PAGE denaturating gel. Band development was done through silver staining as described previously^53^. The samples were in-gel digested with trypsin according to a standard protocol and analysed by Liquid Chromatography Mass Spectrometry (LC-Orbitrap MS/MS) at the MS Facility, SciLifeLab, Uppsala University. The proteins were reduced, alkylated and in-gel digested by trypsin according to a standard operating procedure. Thereafter the samples were dried and resolved in 15 μl 0.1% FA (formic acid). The peptides were separated in reversed-phase on a C18-column and electrosprayed on-line to an LTQ Velos Orbitrap mass spectrometer (Thermo Finnigan). Tandem mass spectrometry was performed applying CID. The database searches were performed using the Sequest algorithm, embedded in Proteome Discoverer 2.1 (ThermoFisher), against human proteins in the Swissprot database (2015). The search criteria for protein identification were set to at least two matching peptides of 95% confidence level per protein.

### Live imaging of hPS cell growth

Human PS cell lines were seeded onto 6-well plates either as small clumps with GCDR or as single cells with TrypLE and 10 μM Y-27632 (no pre-treatment). The imaging was performed with the oCelloscope 04C5 (Unisensor), placed inside a standard normoxic incubator. Pictures were taken from the seeding point and then every 15 min for up to 4 or 5 days (specified for each video file). The imaging was performed using the Uniexplorer 3.2.0.3621 CRL3 software (Unisensor). The exported avi files were further edited using the VideoPad Video Editor (Freeware).

### Statistical analysis

Experiments were performed in at least three independent experiments and in triplicate for each experiment. The data is presented as mean±s.e.m. For the crystal violet cell analysis data multiple statistical analysis was done using one-way ANOVA with Dunnett's post-test, when applicable and stated in the figure legends. Paired *t*-test was used to compare two specific samples within groups, and the result is shown over a line connecting the two samples within the graph. The Human Stem Cell Pluripotency Taqman Array data was analysed using Two-way ANOVA and Sidak's Multiple comparison's test. All these statistical analyses were done using GraphPad Prism version 5.00d for Mac (GraphPad Software), and the data presented shows *P<0.05, **P<0.001 and ***P<0.001 unless otherwise specified. For the copy-number analysis a Wilcoxon rank-sum-test in the statistical software R v3 was used.

### Data availability

Data supporting the findings of this study are available within the article and its Supplementary Information files and from the corresponding author upon reasonable request. The SNP-array genotyping data have been deposited in the NCBI-based Gene Expression Omnibus (GEO) database (http://www.ncbi.nlm.nih.gov/geo/) under the accession code GSE82103.

## Additional information

**How to cite this article:** Pijuan-Galitó, S. *et al.* Human serum-derived protein removes the need for coating in defined human pluripotent stem cell culture. *Nat. Commun.* 7:12170 doi: 10.1038/ncomms12170 (2016).

## Supplementary Material

Supplementary Information Supplementary Figures 1-12 and Supplementary Tables 1-4

Supplementary Data 1 IαI maintains expression profile after long-term passaging. Compilation of dC(t) values from expression profiling using the Human Stem Cell Pluripotency Taqman array. First four columns from the left are hPS cell lines HUES1, H181, H207 and K2C after 15 or more passages in either E8:VN or E8:IαI. Following columns show dC(t) values for all four cell lines after long-term passaging and subsequent spontaneous differentiation through embryoid body formation (EBs). Final columns are dC(t) values for 5 human dermal fibroblasts (HDF) lines. Numbers show C(t) values relative to GAPDH.

Supplementary Movie 1 Human PS cells growth on E8:VN and E8:IαI, small clumps. HUES1 split as small clumps using GCDR and seeded onto Corning 6 well plates on VN-FX coating (E8:VN) and E8:IαI. Cells were imaged from the seeding point and up to 96 hours, with pictures taken every 15 minutes. Medium was changed approximately every 24 hours.

Supplementary Movie 2 Human PS cells growth on E8:VN and E8:IαI, single-cells. HUES1 split as single cells using TrypLE were supplemented with 10 μM ROCKi and seeded onto a Corning 6 well plate, either coated with VN-FX or with the addition of IαI at the seeding step. The cells were imaged from the seeding point and up to 96 hours, images were taken every 15 minutes. Medium was changed approximately every 24 hours.

Supplementary Movie 3 NCL1 forms a colony from one single cell in E8:IαI:ROCKi. NCL1 human ESCs were split as single cells using TrypLE and treated with 10 μM ROCKi. They were seeded onto untreated Corning 6 well plate well with IαI supplementation. The cells were imaged from the seeding point and up to 120 hours, images were taken every 15 minutes. Medium was changed approximately every 24 hours.

Supplementary Movie 4 K2C generates beating cardiomyocytes after E8:IαI culture. K2C human iPSCs were grown for 40 passages in E8:IαI before induced to differentiate through embryoid body formation with 2 weeks of floating culture in 20% FBS medium and subsequent plating for 2 more weeks. Video shows live-time recording of the beating cardiomyocytes using Nikon E990 camera coupled to a bright-field microscope.

## Figures and Tables

**Table 1:** IαI increases cloning efficiency of hPS cells.

**hPS line**	**Culture Method**	**Number of wells**	**Number wells with 1 colony**	**Cloning efficiency (%)**
HUES1	E8:VN	298	7	2.3
HUES1	E8:IαI	451	50	11.1
K2C	E8:VN	100	0	0
K2C	E8:IαI	200	10	5
H207	E8:VN	100	1	1
H207	E8:IαI	93	7	7.5
H181	E8:VN	200	2	1
H181	E8:IαI	200	14	7
K8F	E8:VN	300	29	9.7
K8F	E8:IαI	284	32	11.3
NCL1	E8:IαI	275	50	18.2

Cloning efficiency of individualized HUES1, K2C, H207, H181, K8F and NCL1 hPS cells in 96-well plates using E8:IαI (E8 medium with 50 μg ml^−1^ IαI supplementation) or E8:VN (E8 medium on vitronectin-coated surface) with 5-h pre-treatment and supplementation of 10 μM ROCKi for the first 48 h. Assessment of colonies was done 14 days after seeding through MTT assay.

## References

[b1] Carpenter M. K., Frey-Vasconcells J. & Rao M. S. Developing safe therapies from human pluripotent stem cells. Nat. Biotechnol. 27, 606–613 (2009). 19587662 10.1038/nbt0709-606

[b2] Stojkovic M. *et al.* Derivation of human embryonic stem cells from day-8 blastocysts recovered after three-step *in vitro* culture. Stem Cells 22, 790–797 (2004). 15342943 10.1634/stemcells.22-5-790

[b3] Rodin S. *et al.* Clonal culturing of human embryonic stem cells on laminin-521/E-cadherin matrix in defined and xeno-free environment. Nat. Commun. 5, 3195 (2014). 24463987 10.1038/ncomms4195

[b4] Rodin S. *et al.* Long-term self-renewal of human pluripotent stem cells on human recombinant laminin-511. Nat. Biotechnol. 28, 611–615 (2010). 20512123 10.1038/nbt.1620

[b5] Chen G. *et al.* Chemically defined conditions for human iPSC derivation and culture. Nat. Methods 8, 424–429 (2011). 21478862 10.1038/nmeth.1593 PMC3084903

[b6] Klim J. R., Li L., Wrighton P. J., Piekarczyk M. S. & Kiessling L. L. A defined glycosaminoglycan-binding substratum for human pluripotent stem cells. Nat. Methods 7, 989–994 (2010). 21076418 10.1038/nmeth.1532 PMC2994976

[b7] Melkoumian Z. *et al.* Synthetic peptide-acrylate surfaces for long-term self-renewal and cardiomyocyte differentiation of human embryonic stem cells. Nat. Biotechnol. 28, 606–610 (2010). 20512120 10.1038/nbt.1629

[b8] Villa-Diaz L. G. *et al.* Synthetic polymer coatings for long-term growth of human embryonic stem cells. Nat. Biotechnol. 28, 581–583 (2010). 20512122 10.1038/nbt.1631 PMC3471651

[b9] Brafman D. A. *et al.* Long-term human pluripotent stem cell self-renewal on synthetic polymer surfaces. Biomaterials 31, 9135–9144 (2010). 20817292 10.1016/j.biomaterials.2010.08.007 PMC2949524

[b10] Mei Y. *et al.* Combinatorial development of biomaterials for clonal growth of human pluripotent stem cells. Nat. Mater. 9, 768–778 (2010). 20729850 10.1038/nmat2812 PMC3388774

[b11] Ludwig T. E. *et al.* Feeder-independent culture of human embryonic stem cells. Nat. Methods 3, 637–646 (2006). 16862139 10.1038/nmeth902

[b12] Watanabe K. *et al.* A ROCK inhibitor permits survival of dissociated human embryonic stem cells. Nat. Biotechnol. 25, 681–686 (2007). 17529971 10.1038/nbt1310

[b13] Josic D. *et al.* Proteomic characterization of inter-alpha inhibitor proteins from human plasma. Proteomics 6, 2874–2885 (2006). 16596706 10.1002/pmic.200500563

[b14] Blom A. M., Morgelin M., Oyen M., Jarvet J. & Fries E. Structural characterization of inter-alpha-inhibitor. Evidence for an extended shape. J. Biol. Chem. 274, 298–304 (1999). 9867844 10.1074/jbc.274.1.298

[b15] Atmani F., Glenton P. A. & Khan S. R. Role of inter-alpha-inhibitor and its related proteins in experimentally induced calcium oxalate urolithiasis. Localization of proteins and expression of bikunin gene in the rat kidney. Urol. Res. 27, 63–67 (1999). 10092155 10.1007/s002400050090

[b16] van den Broek I. *et al.* The absolute quantification of eight inter-alpha-trypsin inhibitor heavy chain 4 (ITIH4)-derived peptides in serum from breast cancer patients. Proteomics Clin. Appl. 4, 931–939 (2010). 21137033 10.1002/prca.201000035

[b17] Jayapalan J. J., Ng K. L., Shuib A. S., Razack A. H. & Hashim O. H. Urine of patients with early prostate cancer contains lower levels of light chain fragments of inter-alpha-trypsin inhibitor and saposin B but increased expression of an inter-alpha-trypsin inhibitor heavy chain 4 fragment. Electrophoresis 34, 1663–1669 (2013). 23417432 10.1002/elps.201200583

[b18] Sira M. M., Behairy B. E., Abd-Elaziz A. M., Abd Elnaby S. A. & Eltahan E. E. Serum inter-alpha-trypsin inhibitor heavy chain 4 (ITIH4) in children with chronic hepatitis C: relation to liver fibrosis and viremia. Hepat. Res. Treat. 2014, 307942 (2014). 25295185 10.1155/2014/307942 PMC4177773

[b19] Hamm A. *et al.* Frequent expression loss of Inter-alpha-trypsin inhibitor heavy chain (ITIH) genes in multiple human solid tumors: a systematic expression analysis. BMC Cancer 8, 25 (2008). 18226209 10.1186/1471-2407-8-25 PMC2268946

[b20] Sanggaard K. W. *et al.* The transfer of heavy chains from bikunin proteins to hyaluronan requires both TSG-6 and HC2. J. Biol. Chem. 283, 18530–18537 (2008). 18448433 10.1074/jbc.M800874200

[b21] Hess K. A., Chen L. & Larsen W. J. Inter-alpha-inhibitor binding to hyaluronan in the cumulus extracellular matrix is required for optimal ovulation and development of mouse oocytes. Biol. Reprod. 61, 436–443 (1999). 10411524 10.1095/biolreprod61.2.436

[b22] Garantziotis S. *et al.* Serum inter-alpha-trypsin inhibitor and matrix hyaluronan promote angiogenesis in fibrotic lung injury. Am. J. Respir. Crit. Care. Med. 178, 939–947 (2008). 18703791 10.1164/rccm.200803-386OC PMC2577729

[b23] Werbowetski-Ogilvie T. E. *et al.* Isolation of a natural inhibitor of human malignant glial cell invasion: inter alpha-trypsin inhibitor heavy chain 2. Cancer Res. 66, 1464–1472 (2006). 16452202 10.1158/0008-5472.CAN-05-1913

[b24] Pijuan-Galito S., Tamm C. & Anneren C. Serum Inter-alpha-inhibitor activates the Yes tyrosine kinase and YAP/TEAD transcriptional complex in mouse embryonic stem cells. J. Biol. Chem. 289, 33492–33502 (2014). 25301940 10.1074/jbc.M114.580076 PMC4246103

[b25] Adair J. E. *et al.* Inter-alpha-trypsin inhibitor promotes bronchial epithelial repair after injury through vitronectin binding. J. Biol. Chem. 284, 16922–16930 (2009). 19395377 10.1074/jbc.M808560200 PMC2719329

[b26] Legler D. F., Wiedle G., Ross F. P. & Imhof B. A. Superactivation of integrin alphavbeta3 by low antagonist concentrations. J. Cell. Sci. 114, 1545–1553 (2001). 11282030 10.1242/jcs.114.8.1545

[b27] Yang X. *et al.* cRGD-functionalized, DOX-conjugated, and (6)(4)Cu-labeled superparamagnetic iron oxide nanoparticles for targeted anticancer drug delivery and PET/MR imaging. Biomaterials 32, 4151–4160 (2011). 21367450 10.1016/j.biomaterials.2011.02.006 PMC3292876

[b28] Gil J. E. *et al.* Vitronectin promotes oligodendrocyte differentiation during neurogenesis of human embryonic stem cells. FEBS Lett. 583, 561–567 (2009). 19162023 10.1016/j.febslet.2008.12.061

[b29] Prestwich G. D. Hyaluronic acid-based clinical biomaterials derived for cell and molecule delivery in regenerative medicine. J. Control Release 155, 193–199 (2011). 21513749 10.1016/j.jconrel.2011.04.007 PMC3716467

[b30] Hiraga T., Ito S. & Nakamura H. Cancer stem-like cell marker CD44 promotes bone metastases by enhancing tumorigenicity, cell motility, and hyaluronan production. Cancer Res. 73, 4112–4122 (2013). 23633482 10.1158/0008-5472.CAN-12-3801

[b31] Lokeshwar V. B. *et al.* Antitumor activity of hyaluronic acid synthesis inhibitor 4-methylumbelliferone in prostate cancer cells. Cancer Res. 70, 2613–2623 (2010). 20332231 10.1158/0008-5472.CAN-09-3185 PMC2848908

[b32] Potempa J., Kwon K., Chawla R. & Travis J. Inter-alpha-trypsin inhibitor. Inhibition spectrum of native and derived forms. J. Biol. Chem. 264, 15109–15114 (1989). 2475494

[b33] Ellerstrom C., Strehl R., Noaksson K., Hyllner J. & Semb H. Facilitated expansion of human embryonic stem cells by single-cell enzymatic dissociation. Stem Cells 25, 1690–1696 (2007). 17379766 10.1634/stemcells.2006-0607

[b34] International Stem Cell, I.. *et al.* Characterization of human embryonic stem cell lines by the International Stem Cell Initiative. Nat. Biotechnol. 25, 803–816 (2007). 17572666 10.1038/nbt1318

[b35] Wells J. M. & Melton D. A. Vertebrate endoderm development. Annu. Rev. Cell. Dev. Biol. 15, 393–410 (1999). 10611967 10.1146/annurev.cellbio.15.1.393

[b36] Seguin C. A., Draper J. S., Nagy A. & Rossant J. Establishment of endoderm progenitors by SOX transcription factor expression in human embryonic stem cells. Cell Stem Cell 3, 182–195 (2008). 18682240 10.1016/j.stem.2008.06.018

[b37] Nguyen H. T., Geens M. & Spits C. Genetic and epigenetic instability in human pluripotent stem cells. Hum. Reprod. Update 19, 187–205 (2013). 23223511 10.1093/humupd/dms048

[b38] International Stem Cell, I.. *et al.* Screening ethnically diverse human embryonic stem cells identifies a chromosome 20 minimal amplicon conferring growth advantage. Nat. Biotechnol. 29, 1132–1144 (2011). 22119741 10.1038/nbt.2051 PMC3454460

[b39] Martin M. J., Muotri A., Gage F. & Varki A. Human embryonic stem cells express an immunogenic nonhuman sialic acid. Nat. Med. 11, 228–232 (2005). 15685172 10.1038/nm1181

[b40] Celiz A. D. *et al.* Materials for stem cell factories of the future. Nat. Mater. 13, 570–579 (2014). 24845996 10.1038/nmat3972

[b41] Higuchi A. *et al.* Design of polymeric materials for culturing human pluripotent stem cells: Progress toward feeder-free and xeno-free culturing. Prog. Polym. Sci. 39, 1348–1374 (2014).

[b42] Okroj M. *et al.* Heavy chains of inter alpha inhibitor (IalphaI) inhibit the human complement system at early stages of the cascade. J. Biol. Chem. 287, 20100–20110 (2012). 22528482 10.1074/jbc.M111.324913 PMC3370193

[b43] Carrette O., Nemade R. V., Day A. J., Brickner A. & Larsen W. J. TSG-6 is concentrated in the extracellular matrix of mouse cumulus oocyte complexes through hyaluronan and inter-alpha-inhibitor binding. Biol. Reprod. 65, 301–308 (2001). 11420253 10.1095/biolreprod65.1.301

[b44] Zhang S., He H., Day A. J. & Tseng S. C. Constitutive expression of inter-alpha-inhibitor (IalphaI) family proteins and tumor necrosis factor-stimulated gene-6 (TSG-6) by human amniotic membrane epithelial and stromal cells supporting formation of the heavy chain-hyaluronan (HC-HA) complex. J. Biol. Chem. 287, 12433–12444 (2012). 22351758 10.1074/jbc.M112.342873 PMC3320993

[b45] Shay E., He H., Sakurai S. & Tseng S. C. Inhibition of angiogenesis by HC.HA, a complex of hyaluronan and the heavy chain of inter-alpha-inhibitor, purified from human amniotic membrane. Invest. Ophthalmol. Vis. Sci. 52, 2669–2678 (2011). 21228375 10.1167/iovs.10-5888 PMC3088557

[b46] Wisniewski H. G., Burgess W. H., Oppenheim J. D. & Vilcek J. TSG-6, an arthritis-associated hyaluronan binding protein, forms a stable complex with the serum protein inter-alpha-inhibitor. Biochemistry 33, 7423–7429 (1994). 7516184 10.1021/bi00189a049

[b47] Mould A. P., Akiyama S. K. & Humphries M. J. The inhibitory anti-beta1 integrin monoclonal antibody 13 recognizes an epitope that is attenuated by ligand occupancy. Evidence for allosteric inhibition of integrin function. J. Biol. Chem. 271, 20365–20374 (1996). 8702772 10.1074/jbc.271.34.20365

[b48] Hovatta O. *et al.* A culture system using human foreskin fibroblasts as feeder cells allows production of human embryonic stem cells. Hum. Reprod. 18, 1404–1409 (2003). 12832363 10.1093/humrep/deg290

[b49] Enghild J. J., Thogersen I. B., Pizzo S. V. & Salvesen G. Analysis of inter-alpha-trypsin inhibitor and a novel trypsin inhibitor, pre-alpha-trypsin inhibitor, from human plasma. Polypeptide chain stoichiometry and assembly by glycan. J. Biol. Chem. 264, 15975–15981 (1989). 2476436

[b50] Miyazaki T. *et al.* Laminin E8 fragments support efficient adhesion and expansion of dissociated human pluripotent stem cells. Nat. Commun. 3, 1236 (2012). 23212365 10.1038/ncomms2231 PMC3535336

[b51] Sturn A., Quackenbush J. & Trajanoski Z. Genesis: cluster analysis of microarray data. Bioinformatics 18, 207–208 (2002). 11836235 10.1093/bioinformatics/18.1.207

[b52] Meisner L. F. & Johnson J. A. Protocols for cytogenetic studies of human embryonic stem cells. Methods 45, 133–141 (2008). 18593610 10.1016/j.ymeth.2008.03.005

[b53] Shevchenko A., Wilm M., Vorm O. & Mann M. Mass spectrometric sequencing of proteins silver-stained polyacrylamide gels. Anal. Chem. 68, 850–858 (1996). 8779443 10.1021/ac950914h

