# Supplementary material for: Human serum-derived protein removes the need for coating in defined human pluripotent stem cell culture
Source: Nat Commun. 2016 Jul 13;7:12170. doi: 10.1038/ncomms12170 (PMC4947164; doi:10.1038/ncomms12170)
Supplement: Supplementary Information — Supplementary Figures 1-12 and Supplementary Tables 1-4 [file ncomms12170-s1.pdf]

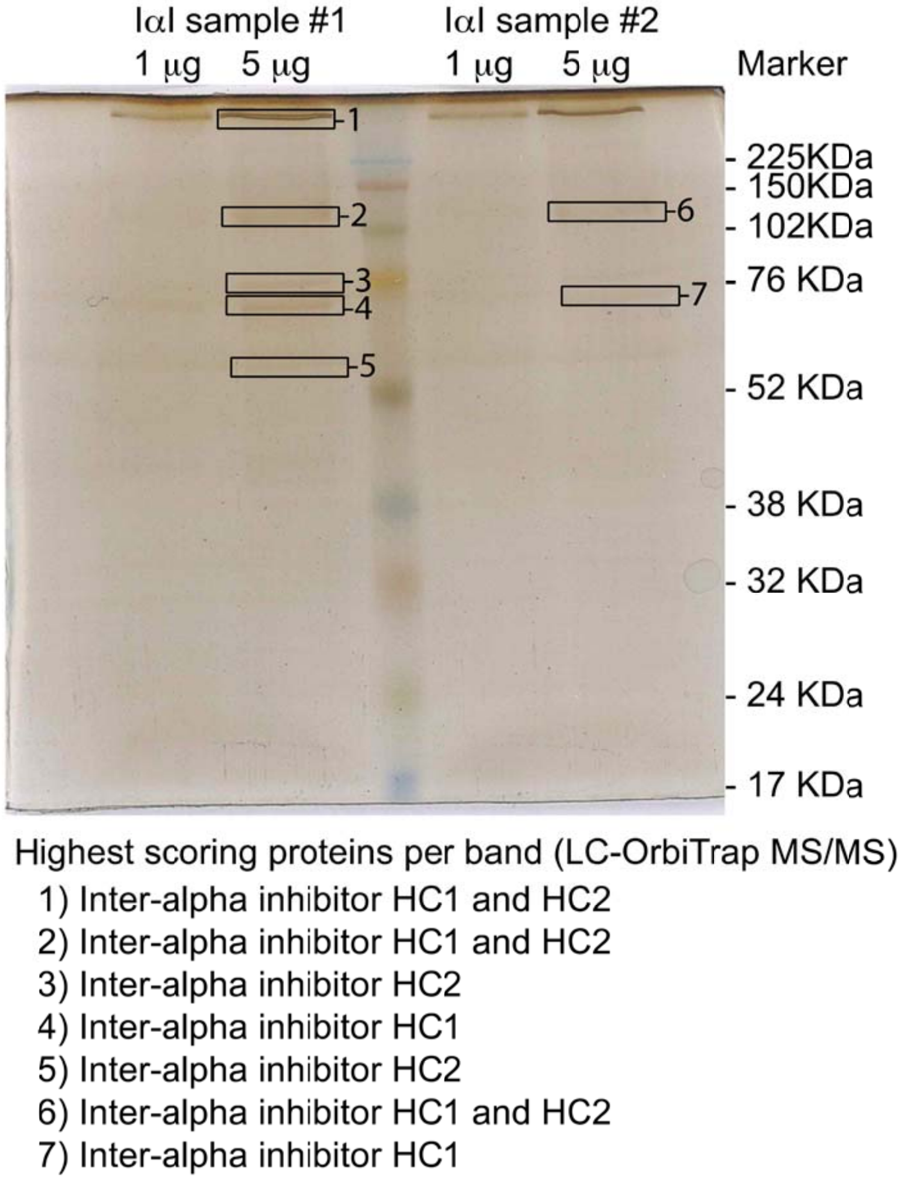

Supplementary Figure 1: Purified IαI does not contain any major impurities. Two separate samples of purified Inter-α-inhibitor (IαI) containing 1 or 5 μg total protein were run on a denaturing 12% acrylamide SDS-PAGE gel and subjected traditional silver staining. All major protein bands (indicated by numbered boxes) were excised and identified through LC-Orbitrap MS/MS (Liquid Chromatography Mass Spectrometry). The highest scoring proteins for each band are listed below.

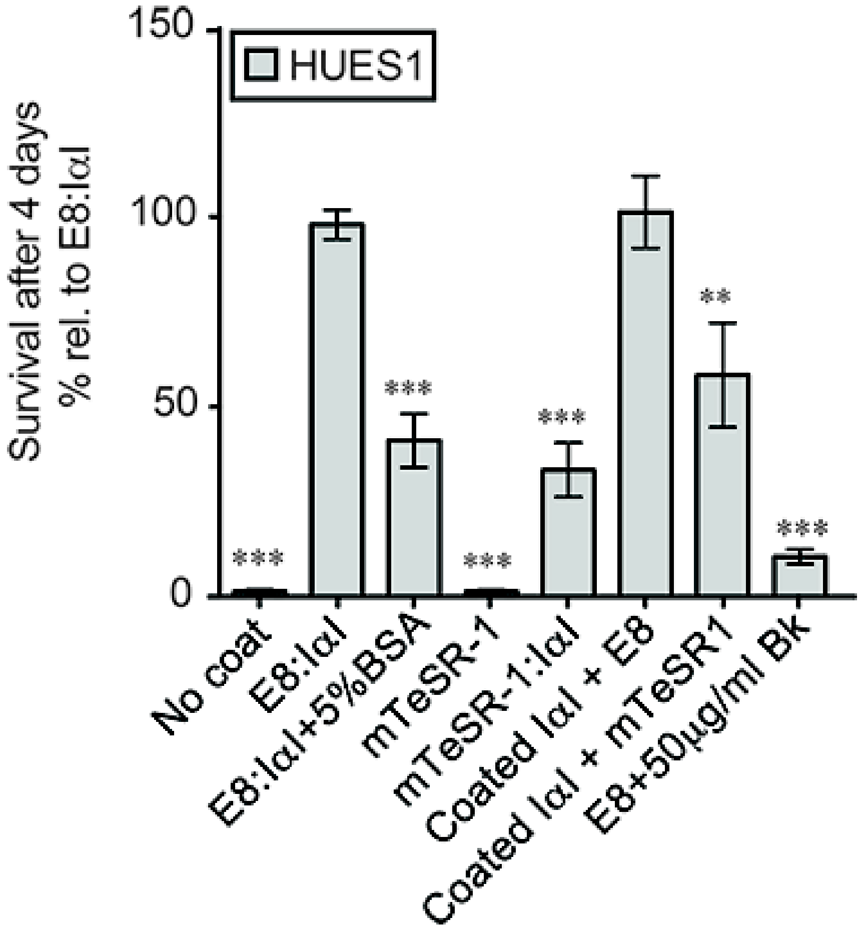

Supplementary Figure 2: Albumin hinders IαI-mediated attachment. Cell survival and growth assayed with crystal violet staining after 4 days of HUES1 human pluripotent stem (hPS) cell culture in E8 medium with or without 50 μg ml^{-1} IαI (E8:IαI), 5% bovine serum albumin (BSA, w/v) in E8: IαI, mTeSR1 medium with or without IαI, coated 50 μg ml^{-1} IαI and E8 medium, coated IαI with mTeSR1 medium or with the supplement of 50 μg ml^{-1} bikunin (Bk) on uncoated tissue culture treated (TC)-treated plastic. Bars show mean ± SEM and statistical analysis was performed using One-way ANOVA and Dunnett's post-test against positive control E8:IαI (GraphPad Prism) and indicates significant differences with * = p<0.05, ** = p<0.001 and *** = p<0.001.

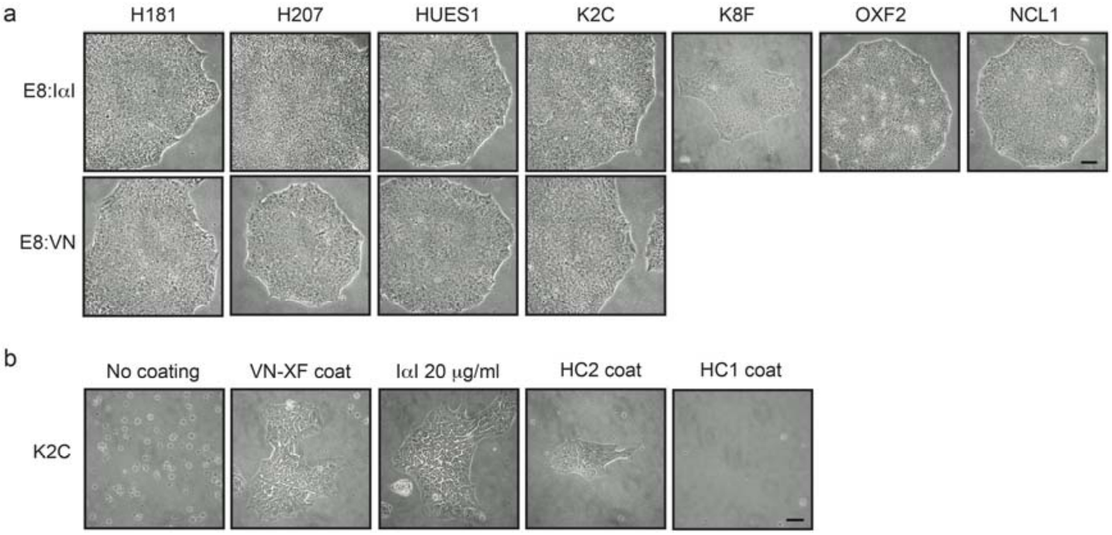

Supplementary Figure 3: IαI and its domain HC2 support attachment of hPS cells. (a) Representative brightfield images of hPS cell lines HUES1, K2C, H207, H181, K8F, OXF2 and NCL1 cultured in E8 medium with 50 μg ml^{-1} IαI supplementation (E8:IαI), and on vitronectin coating peptide (E8:VN); (b) Brightfield images of hiPS cell line K2C 4 days after seeding in E8 medium on non-coated plastic, vitronectin peptide (VN-FX) coating, 20 μg ml^{-1} IαI supplementation, 20 μg ml^{-1} HC2 coating and 20 μg ml^{-1} HC1 coating. Scale bar shows 100μm.

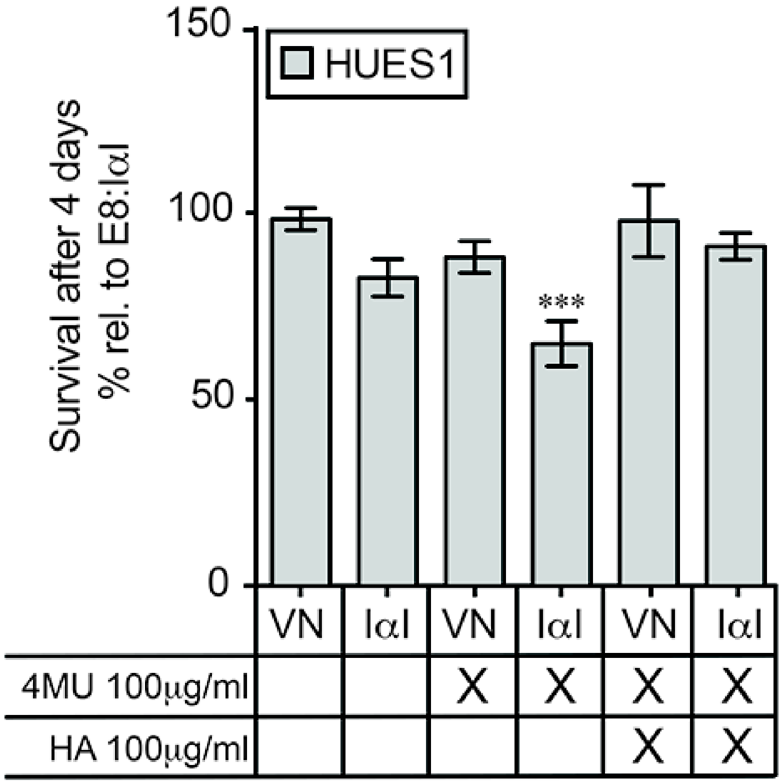

Supplementary Figure 4: GAG synthesis inhibition decreases IαI-mediated attachment. Cell survival and growth assayed with crystal violet staining after 4 days of HUES1 hES cell culture in E8 medium on VN coating or with IαI supplementation with and without 0.6 mM (100 μg ml^{-1}) 4-metyhlumbelliferone (4-MU)-treatment and with the addition or not of 100 μg ml^{-1} of HMW-HA (high molecular weight Hyaluronan, 2000-2400 KDa) for the first 24 hours after seeding. Bars show mean ± SEM and statistical analysis was performed using One-way ANOVA and Dunnett's post-test against positive control E8 with VN coating (GraphPad Prism) and indicates significant differences with * = p<0.05, ** = p<0.001 and *** = p<0.001.

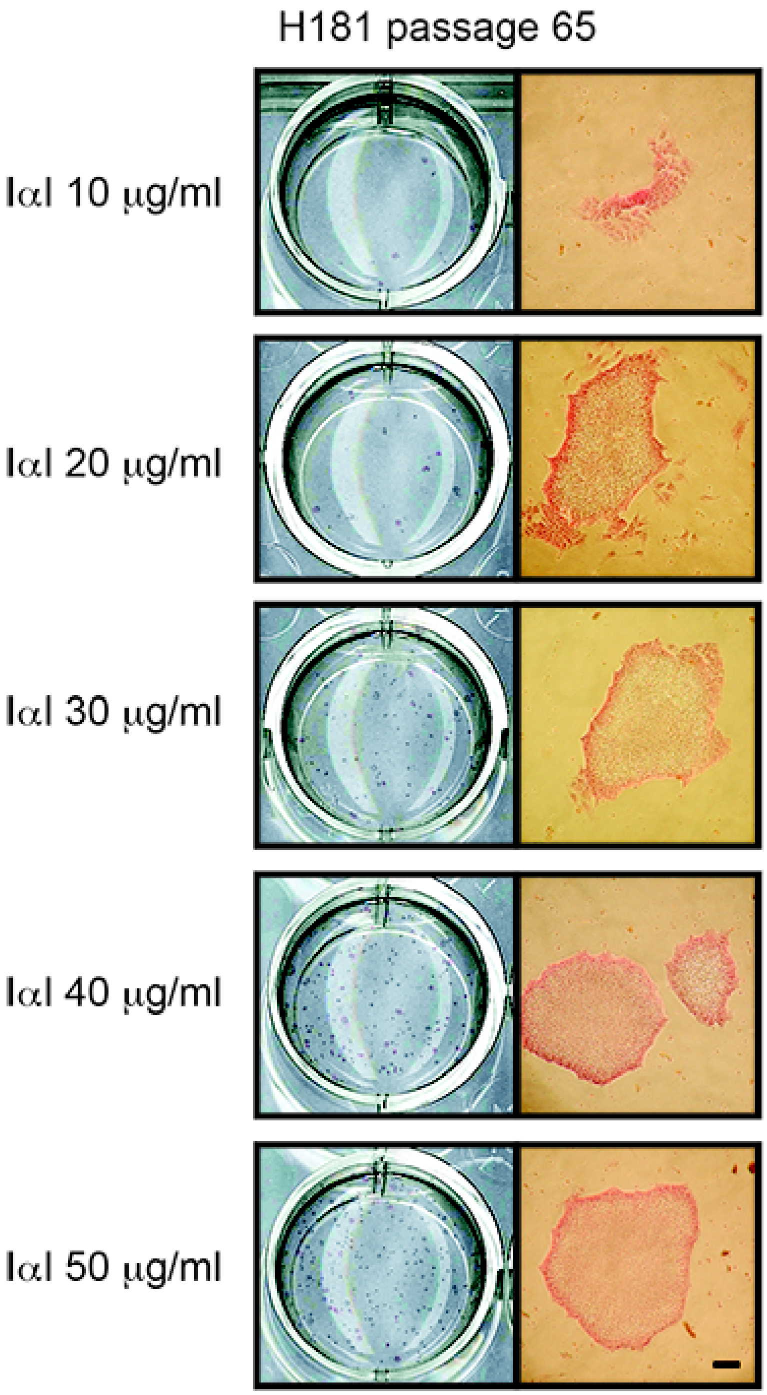

Supplementary Figure 5: IαI supports pluripotency of hPS cells. Brightfield images of alkaline phosphatase staining of hES cell line H181 four days after seeding with increasing concentrations of IαI supplemented to the medium at the seeding step. Scale bar shows 100μm.

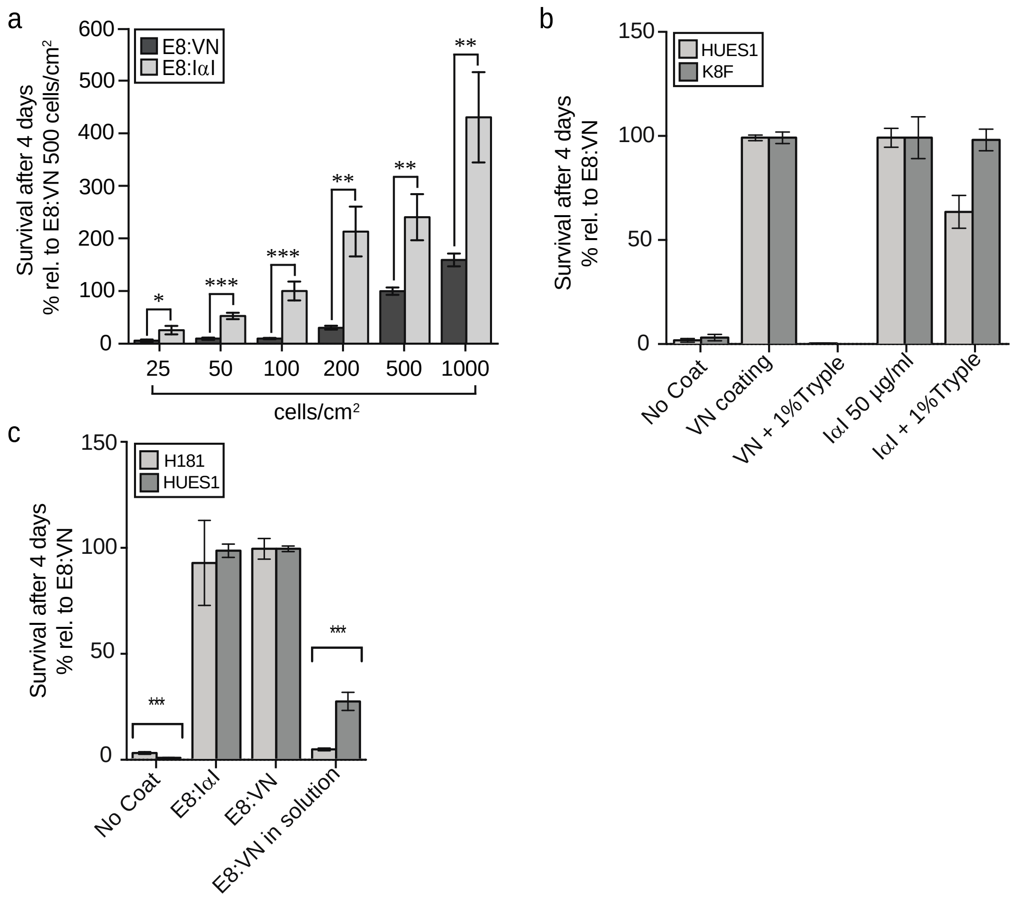

Supplementary Figure 6: IαI increases single-cell survival over VN-FX. Cell density after single cell splitting of HUES1 hES cell line as single cells using TrypLE with 5 hour 10μM ROCKi (Rho-associated kinase inhibitor, Y-27632) pre-treatment and supplementation for the first 24 hours a) seeded in E8:VN or E8:IαI using increasing seeding cell densities b) seeded at density $5x10^3$ cells cm^{-2} in E8:VN or E8:IαI with and without the addition of 1% TrypLE at the seeding step and c) seeded at density $5x10^3$ cells cm^{-2} in E8: VN (coated), E8:IαI or in E8 supplemented with 10 μg ml^{-1} VN peptide (no coating). All cell number quantification experiments were performed in triplicate over three separate experiments except for 6b, which was done only in two independent experiments, and therefore shows no statistical analysis. Bars show mean ± SEM and statistical analysis indicates significant differences with * = p<0.05, ** = p<0.001 and *** = p<0.001 in (a) paired t-test between each seeding condition for each cell density seeding and (c) One-way ANOVA and Dunnett's post-test multiple comparison to VN coating control values.

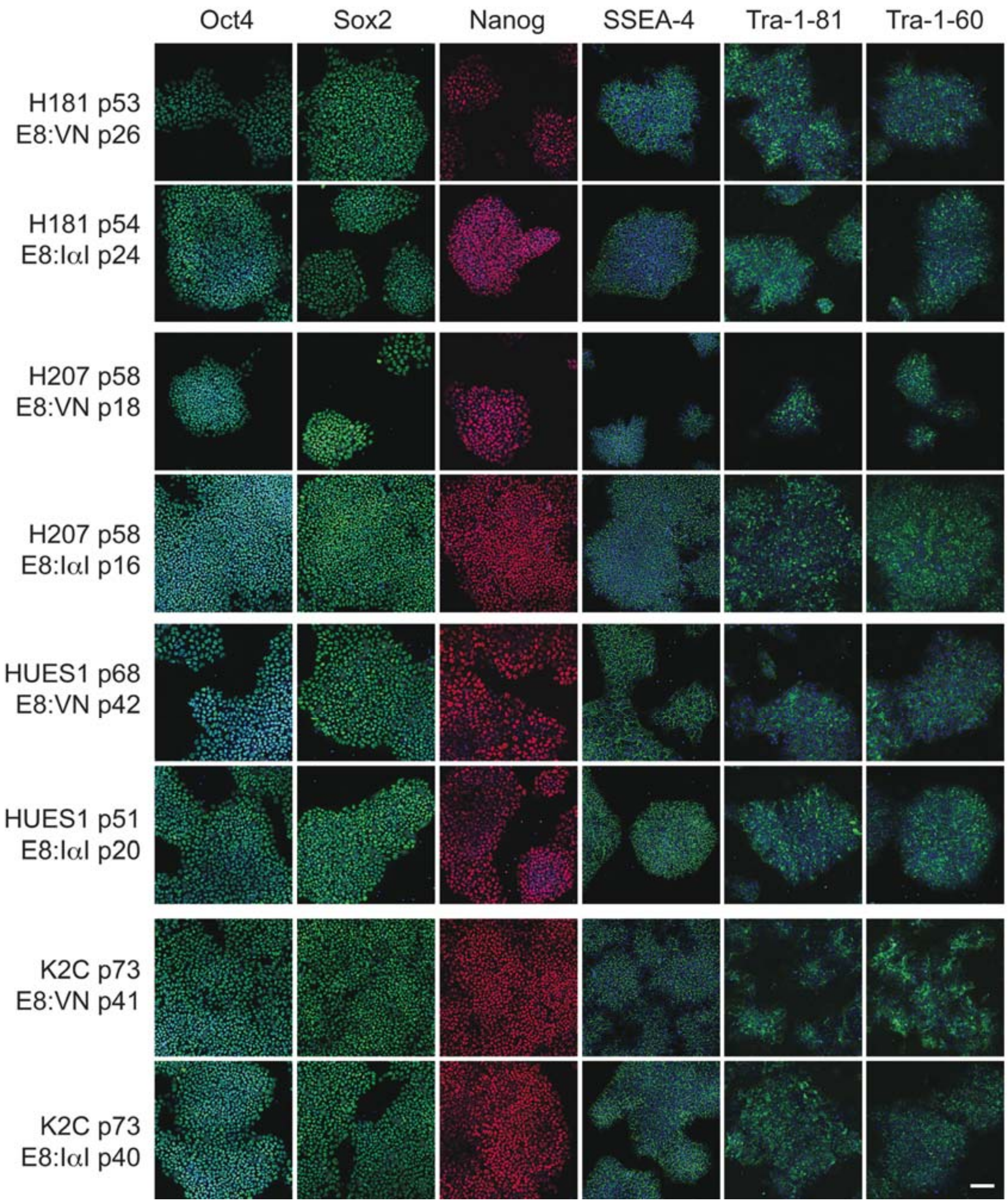

Supplementary Figure 7: IαI maintains stem cell markers in continuous culture.

Immunofluorescent staining for stem cell markers Oct4 (green), Sox2 (green), Nanog (red), SSEA-4 (green), Tra-1-81 (green), Tra-1-60 (green), together with DAPI nuclear staining (blue) in the four hPS cell lines HUES1, K2C, H181 and H207 after 15 or more passages in E8:VN and E8:IαI. Scale bar represents 100μm.

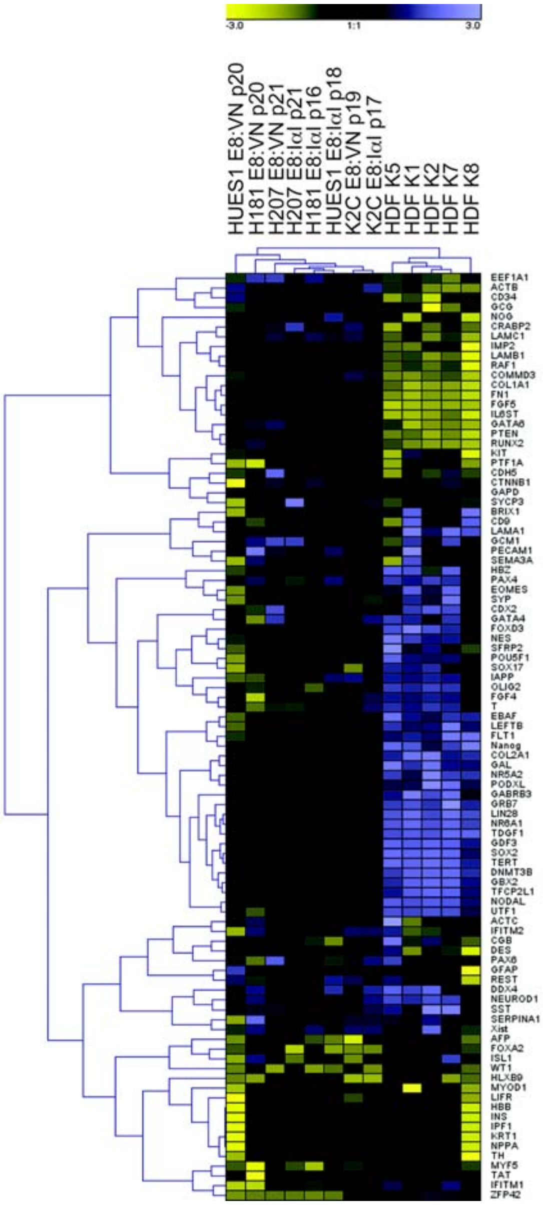

Supplementary Figure 8: IαI maintains RNA expression profile of hPS cells. Two-way hierarchical cluster analysis (Genesis) using dC(t) values relative to GAPDH from a Taqman pluripotency array for hPS cell lines HUES1, H181, H207 and K2C after 15 or more passages in either E8:VN or E8:IαI in comparison to five different samples of human dermal fibroblasts (HDFs). Expression levels are related to the median for each transcript and expression is illustrated in color from blue (high dC(t), low expression) to yellow (low dC(t), high expression). Note that E8:IαI culture conditions cluster more closely than E8:VN.

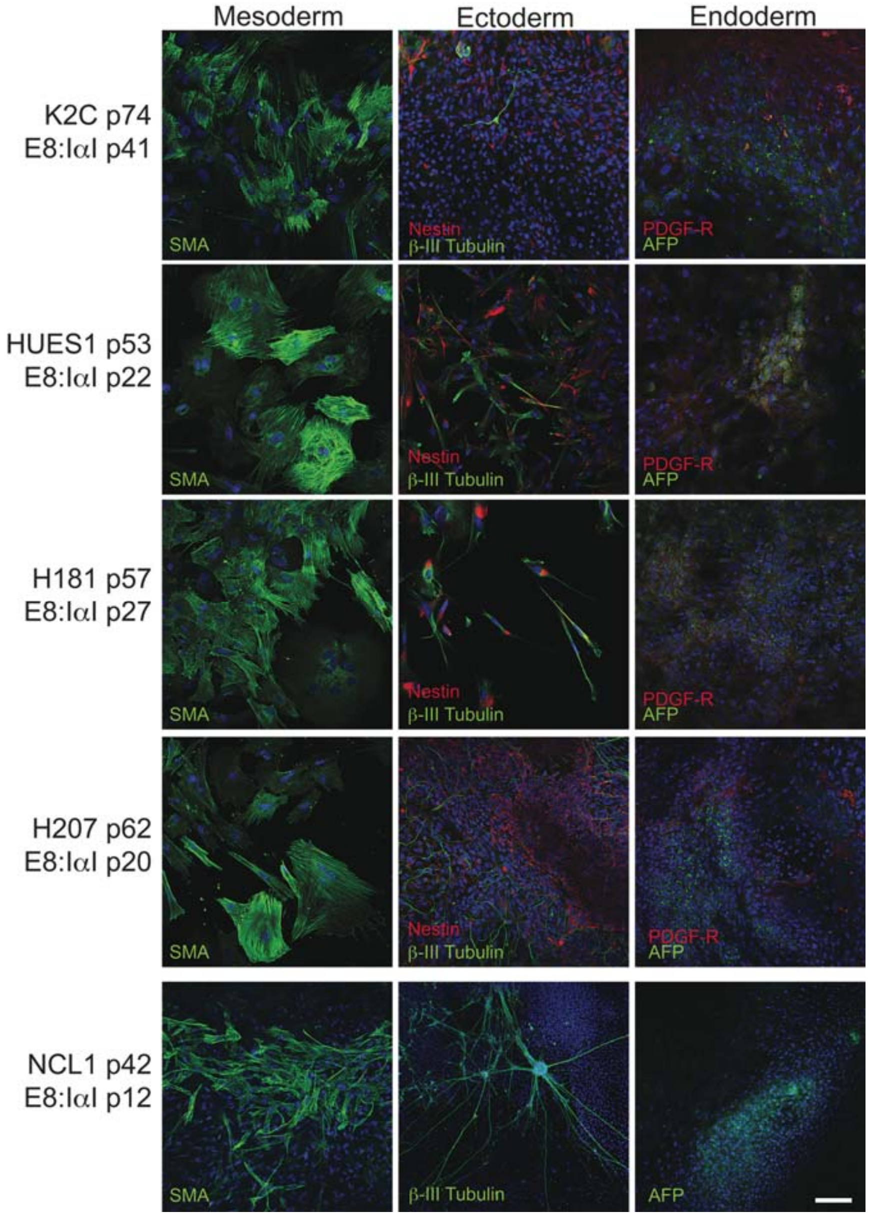

Supplementary Figure 9: IαI maintains pluripotency after long-term passaging.

Immunocytochemistry of differentiated hPS cell lines after 15 or more passages in E8:IαI. Human PS cells were harvested and allowed for embryoid body (EB) formation, the EBs were maintained in suspension in 20% serum medium for 2 weeks following TrypLE dissociation and plating onto matrigel where they were allowed to differentiate for 2 weeks. Immunofluorescent staining for markers of the 3 different germ layers: Ectoderm (Nestin in red and β-III-tubulin in green), Mesoderm (α-smooth muscle actin, αSMA, green) and Endoderm (alpha-fetoprotein, AFP, in green and PDGF-receptor in red), together with DAPI nuclear staining (blue). Scale bar shows 100μm.

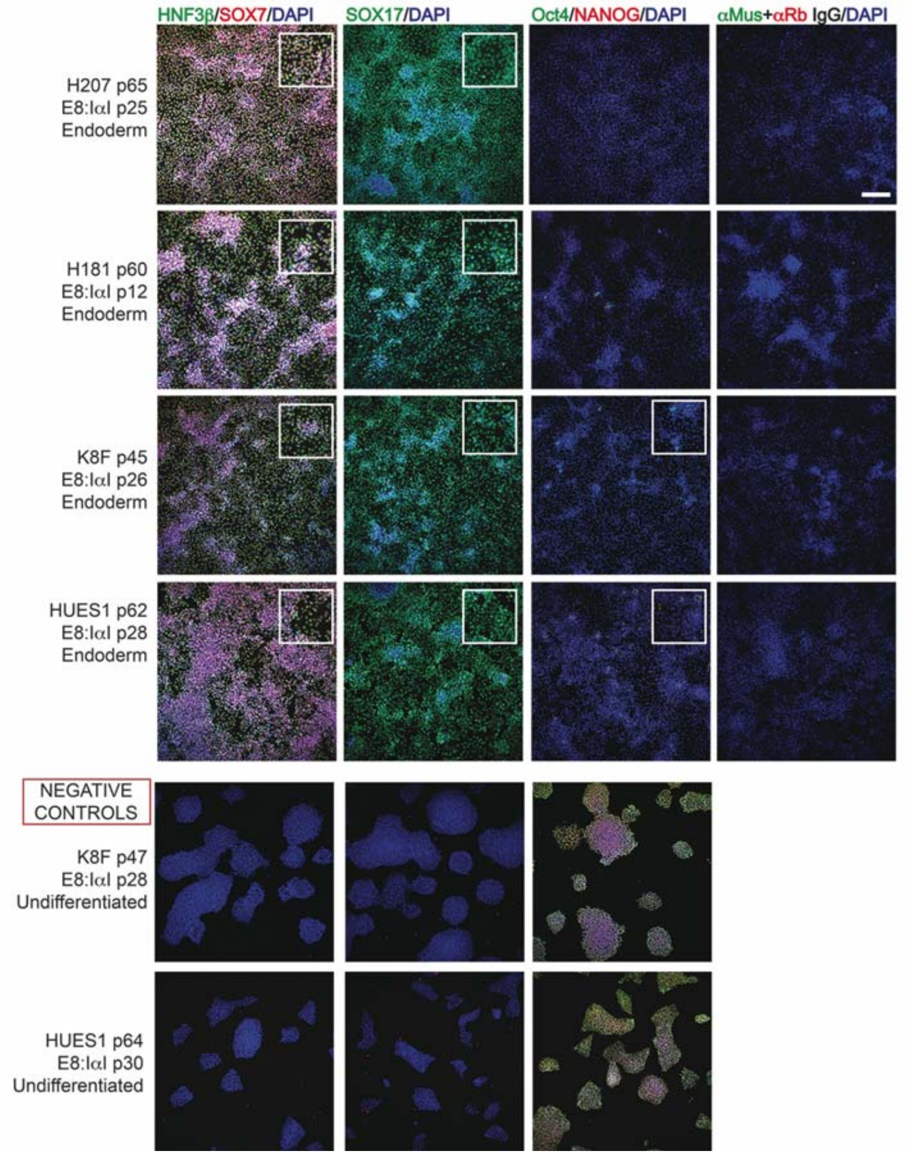

Supplementary Figure 10: IαI supports directed endoderm differentiation. Four hPS cell lines were differentiated into early endoderm using the StemDiff Endoderm Differentiation Kit (StemCell Technologies). Briefly, hPS cells grown in E8:IαI were conditioned to endoderm and seeded using IαI supplement instead of VN coating. Results show immunocytochemistry of differentiated hPS cell after a 5-day differentiation protocol for the endoderm markers Sox7 (red), Sox17 (green) and HNF3β (green), and the control pluripotency markers Nanog (red) and Oct4 (green), as well as negative control using only labeled secondary antibodies, in combination with DAPI nuclear staining. Negative controls were also performed to ensure proper antibody specificity using undifferentiated HUES1 and K8F hPS cells. White box shows close-up image and scale bar shows 100μm.

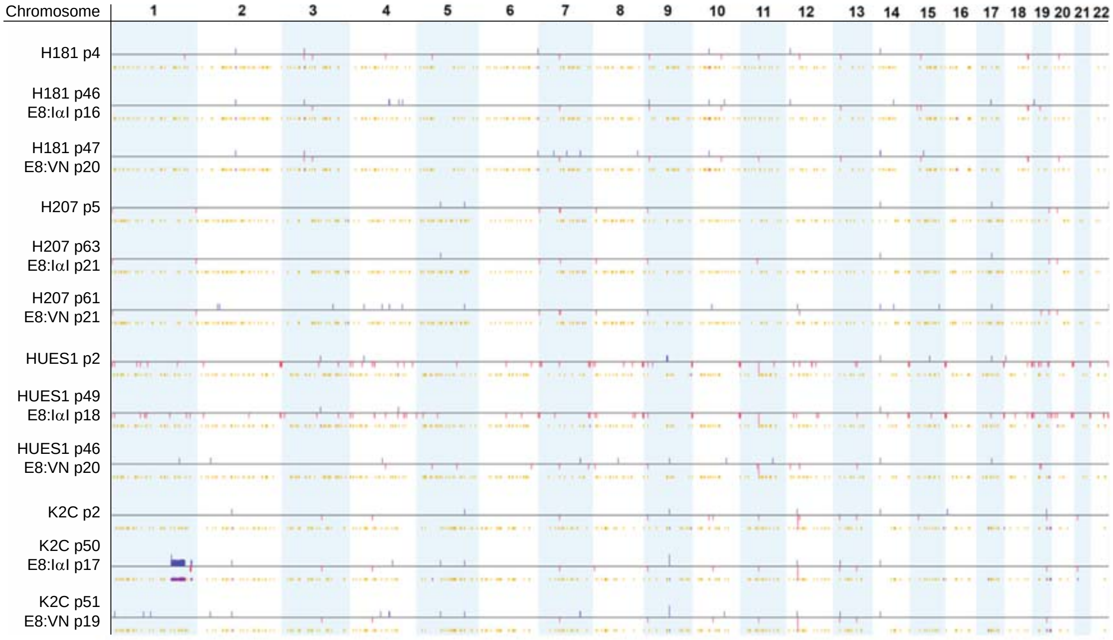

Supplementary Figure 11: E8:IαI does not increase genetic modifications. Results from 12 copy-number (CN) profiling experiments using Illumina's HumanOmniExpressExome-array, encompassing >964,000 single nucleotide polymorphism (SNP)-markers. The CN-calls of the autosomal chromosomes are illustrated using the Nexus-software. Each of the four hPS cell lines (i.e. H181, H207, HUES1 and K2C) was analyzed in three separate SNP-array experiments. A first analysis was performed after 2-5 passages of growth for initial reference, and subsequently, two analyses were performed per cell line after 16-21 passages in E8:VN or E8:IαI culture. The cell lines, treatment (IαI or VN) as well as passage number (2-21) for each experiment is indicated for each sample. Blue = CN-gain; red = CN-loss; yellow = loss of heterozygosity (LOH).

H181 E8:IαI
46,XX,der(4)t(4;18)(p16;q11)

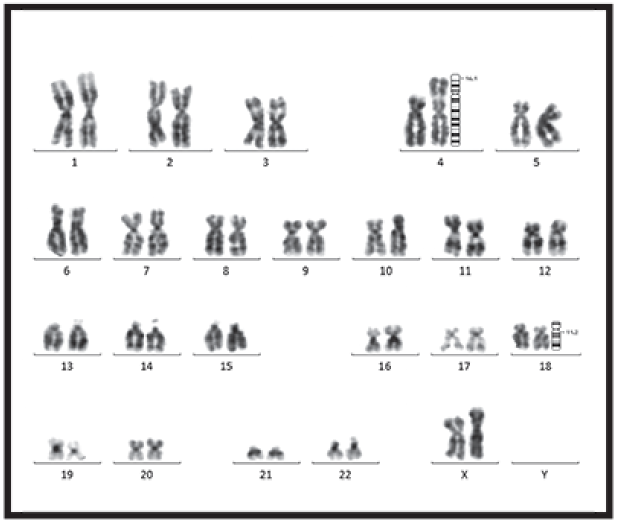

K2C E8:IαI
46,XY,dup(1)(q32q42)

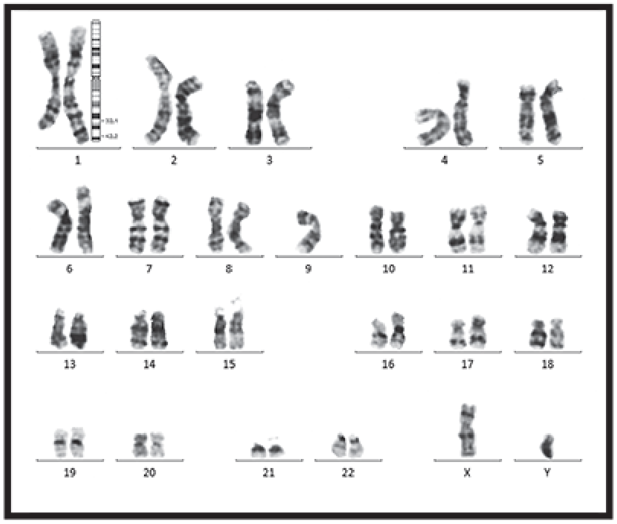

Supplementary Figure 12: E8:IαI does not induce increased chromosomal aberrations.

Representative images of the chromosomal aberrations found in H181 and K2C hPS cell lines after long-term passage in E8:IαI.

Supplementary Table 1: Description of integrin blocking peptides (Bachem).

Cat. No.	PEPTIDE DESCRIPTION
**H-1830**	**RGD:** Cell adhesion motif shared by many extracellular matrix proteins, such as VN, laminin (LN) and von Willebrand factor. (Mw: 346.34 Da)
**H-4088**	**c(RADfV):** Control peptide for H-2574. (Mw: 588.66 Da)
**H-2574**	**c(RGDfV):** Binds $a_v\beta_3$ and $\alpha_v\beta_5$ integrin receptors. Studies show effects inhibiting tumor cell adhesion to LN and VN and *in vivo* preventing cell-cell adhesion in rats, inhibiting tubular obstruction in the kidney[47, 48]. (Mw: 574.64 Da)
**H-7232**	**c(RADfC):** Control peptide for H-7226. (Mw: 592.68 Da)
**H-7226**	**c(RGDfC):** RGD tumor-targeting peptide, binds avβ3 receptors[49]. (Mw: 578.64 Da)
**H-3164**	**GRGDsP**: Inhibitor of cell attachment to fibronectin but not VN[50]. (Mw: 587.59 Da)

Supplementary Table 2: IαI does not increase karyotype abnormalities.

Cell line & Culture method	G-banding analysis
**K2C E8:VN**	46,XY[20]
**K2C E8:IαI**	46,XY,dup(1)(q32q42)[1]/46,XY[19]
**H207 E8:VN**	46,XY[20]
**H207 E8:IαI**	46,XY[20]
**H181 E8:VN**	47,XX+20[12]/46,XX[8]
**H181 E8:IαI**	46,XX,der(4)t(4;18)(p16;q11)[2]/46,XX[18]
**HUES1 E8:VN**	46,XX[20]
**HUES1 E8:IαI**	46,XX[20]

Karyotyping analysis of hiPS cell line K2C and hES cell lines H207, H181 and HUES1 after 15 or more passages in either E8:VN or E8:IαI.

Supplementary Table 3: Total Copy-number variation analysis.

**Cell line**	**Passage**	**Protocol**	**Total CN aberrations**	**% ratio of CN-calls in later passage compared to CN-calls in early passage (%)**
**H181**	4		199	
**H181**	16	E8:IαI	32	**16.1**
**H181**	20	E8:VN	31	**15.6**
**H207**	5		138	
**H207**	21	E8:IαI	32	**23.2**
**H207**	21	E8:VN	91	**65.9**
**HUES1**	2		269	
**HUES1**	18	E8:IαI	137	**50.9**
**HUES1**	20	E8:VN	35	**13.0**
**K2C**	2		161	
**K2C**	17	E8:IαI	179	**111.2**
**K2C**	19	E8:VN	109	**67.7**

Total number of copy-number (CN)-calls found in each of the four hPS cell lines in the early passages just after E8 medium adaptation, and 15 or more passages after long-term growth in each protocol (E8:IαI or E8:VN). Furthest right column shows percentage ratio of shared (CN)-calls between early and later passages.

Supplementary Table 4: Copy-number variation analysis as compared to starting culture.

Cell line/Culture method	Total	CN-loss	CN-gain	Shared CN-calls
**H181 E8:IαI**	11	7	4	34
**H181 E8:VN**	14	9	5	45
**H207 E8:IαI**	10	7	3	31
**H207 E8:VN**	10	8	2	11
**HUES1 E8:IαI**	45	43	2	33
**HUES1 E8:VN**	8	6	2	23
**K2C E8:IαI**	13	10	3	7
**K2C E8:VN**	12	9	3	11

Number of CN-call variation on end-point hPS cell culture in each condition when compared to the starting early passages. Table shows total CN-calls that changed over the long-term passage in each hPS cell line and condition (Total), CN-calls lost compared to early passage (CN-loss), CN-calls gained compared to early passage (CN-gain), and number of shared CN-calls between early and late passage (Shared CN-calls).
